# Advances in Inflammatory Bowel Disease Diagnostics: Machine Learning and Genomic Profiling Reveal Key Biomarkers for Early Detection

**DOI:** 10.3390/diagnostics14111182

**Published:** 2024-06-04

**Authors:** Asif Hassan Syed, Hamza Ali S. Abujabal, Shakeel Ahmad, Sharaf J. Malebary, Nashwan Alromema

**Affiliations:** 1 Department of Computer Science, Faculty of Computing and Information Technology-Rabigh, King Abdulaziz University, Jeddah 22254, Saudi Arabia; sarahmad@kau.edu.sa; 2 Department of Mathematics, Faculty of Science, King Abdulaziz University, P.O. Box 80203, Jeddah 21589, Saudi Arabia; prof.h.abujabal@gmail.com; 3 Department of Information Technology, Faculty of Computing and Information Technology-Rabigh, King Abdulaziz University, P.O. Box 344, Rabigh 21911, Saudi Arabia; smalebary@kau.edu.sa; 4 Department of Computer Science, Faculty of Computing and Information Technology-Rabigh, King Abdulaziz University, P.O. Box 344, Rabigh 21911, Saudi Arabia; nalromema@kau.edu.sa

**Keywords:** inflammatory bowel disease (IBD), high-throughput technologies, differentially expressed genes (DEGs), machine learning (ML), feature selection (FS), gene ontology, pathway enrichment analysis

## Abstract

This study, utilizing high-throughput technologies and Machine Learning (ML), has identified gene biomarkers and molecular signatures in Inflammatory Bowel Disease (IBD). We could identify significant upregulated or downregulated genes in IBD patients by comparing gene expression levels in colonic specimens from 172 IBD patients and 22 healthy individuals using the GSE75214 microarray dataset. Our ML techniques and feature selection methods revealed six Differentially Expressed Gene (DEG) biomarkers (*VWF*, *IL1RL1*, *DENND2B*, *MMP14*, *NAAA*, and *PANK1*) with strong diagnostic potential for IBD. The Random Forest (RF) model demonstrated exceptional performance, with accuracy, F1-score, and AUC values exceeding 0.98. Our findings were rigorously validated with independent datasets (GSE36807 and GSE10616), further bolstering their credibility and showing favorable performance metrics (accuracy: 0.841, F1-score: 0.734, AUC: 0.887). Our functional annotation and pathway enrichment analysis provided insights into crucial pathways associated with these dysregulated genes. *DENND2B* and *PANK1* were identified as novel IBD biomarkers, advancing our understanding of the disease. The validation in independent cohorts enhances the reliability of these findings and underscores their potential for early detection and personalized treatment of IBD. Further exploration of these genes is necessary to fully comprehend their roles in IBD pathogenesis and develop improved diagnostic tools and therapies. This study significantly contributes to IBD research with valuable insights, potentially greatly enhancing patient care.

## 1. Introduction

### 1.1. Background

Inflammatory Bowel Disease (IBD), which encompasses ulcerative colitis (UC) and Crohn’s disease (CD), is a chronic inflammatory condition that affects the gastrointestinal tract. It has a significant global impact, affecting millions worldwide [1,2]. Early and accurate detection of IBD is crucial for effective disease management and personalized treatment, but the complex and heterogeneous nature of IBD poses diagnostic challenges [3].

### 1.2. Research Motivation

Advancements in high-throughput transcriptomic microarray technologies have provided opportunities to explore gene expression profiles associated with IBD. These datasets offer insights for identifying diagnostic biomarkers to distinguish IBD patients from healthy individuals [4]. However, analyzing high-dimensional low-sample size (HDLSS) transcriptomic data remains challenging [5,6]. Machine learning (ML) techniques have emerged as powerful tools for analyzing complex biological datasets and discovering predictive patterns [7,8,9].

### 1.3. An Overview of the Study Objectives and Methodology

This study explores high-throughput technologies and ML to identify molecular signatures associated with IBD, enhancing our understanding of IBD pathogenesis [10,11,12,13,14,15,16]. We aim to employ supervised feature selection (FS) methods to identify informative gene biomarkers for accurately classifying IBD patients and healthy controls, facilitating earlier diagnosis and personalized treatment.

The main goals of this study are as follows:

1. Evaluate the effectiveness of high-throughput technologies and ML in identifying molecular signatures and enhancing our understanding of IBD pathogenesis.
2. Assess the accuracy and reliability of the identified gene biomarkers for diagnosis of IBD.
3. Investigate the impact of the identified gene biomarkers on IBD diagnosis and personalized treatment.

We have devised an ML-based framework to achieve these goals using a publicly available transcriptomic microarray dataset (GEO75214) from the GEO database [17]. This dataset will be used to discover DEGs associated with IBD. We will employ a comprehensive set of supervised FS approaches and various visualization tools to analyze the DEGs and identify the most informative genes associated with IBD. The selected DEGs will be utilized to train a set of supervised-learning classifiers, and their performance will be thoroughly evaluated using relevant metrics such as AUC-ROC and accuracy [18].

Furthermore, we validate the identified gene biomarkers using independent cohorts from the GEO database (GEO10616 and GEO36807). These validation cohorts assess the reliability and applicability of the identified biomarkers. Additionally, we perform gene ontology (GO) and pathway enrichment analysis on the identified DEGs using the Overrepresentation Enrichment Analysis (ORA) method available through the WebGestalt toolkit 2024 [19]. This analysis provides insights into the molecular mechanisms, disrupted biological pathways, and processes associated with IBD.

### 1.4. Main Contributions

The study contributes to the field of IBD research in the following ways:

- Discovery of novel gene biomarkers: the study identifies *DENND2B* and *PANK1* as novel biomarkers with strong diagnostic potential for IBD.
- Validation of biomarkers: the study rigorously validates the identified gene biomarkers using independent datasets, confirming their reliability and generalizability.
- Enhanced understanding of IBD pathogenesis: the study improves our understanding of the molecular mechanisms and disrupted pathways associated with IBD.
- Facilitated early detection: the study develops a diagnostic model based on the identified biomarkers, enabling accurate and timely detection of IBD.
- Personalized treatment approaches: the study’s findings provide insights for tailoring treatment plans based on individual patients’ IBD subtypes and disease severity.

Through these contributions, this study makes significant strides in IBD research. The discovery of novel gene biomarkers, their validation, enhanced understanding of IBD pathogenesis, and the potential for early detection and personalized treatment approaches collectively contribute to the advancement of knowledge and the potential for improved patient care in the field of IBD. Subsequent sections of the research paper present detailed literature reviews, providing comprehensive insights into the existing body of knowledge in the field.

**Figure 1:**
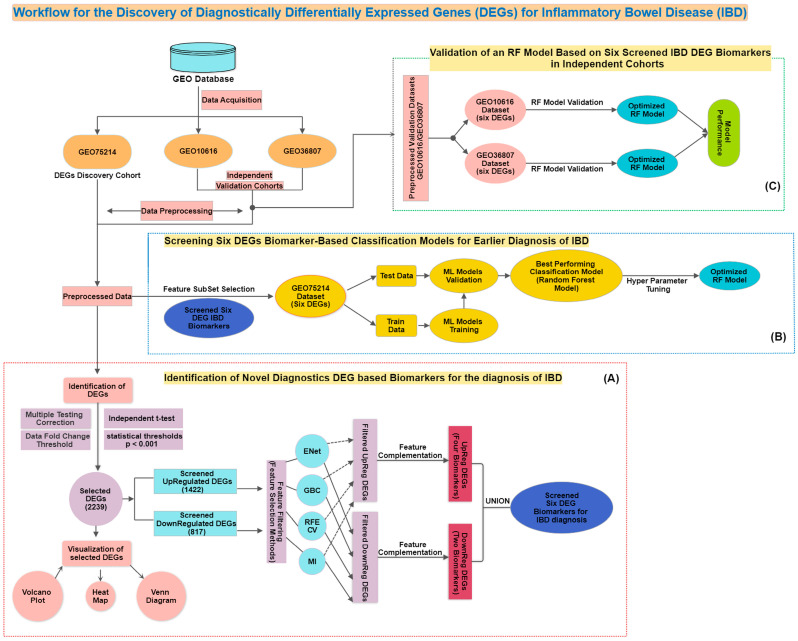
(**A**) Illustrates the intended framework for selecting and identifying potential DEGs from the GEO75214 gene expression dataset. (**B**) Depicts the framework s to screen the best supervised classification model that effectively differentiates IBD from healthy control samples. (**C**) Represents the RF model built using the six DEG biomarkers in independent cohorts.

The research article follows a structured outline. Section 1 introduces the research topic, outlines the study’s objectives, and provides an overview of the contributions made by the research work. Section 2 presents a comprehensive review of the existing research on identifying gene biomarkers associated with IBD. In Section 3, the methodology employed in the research paper is described, including the dataset description, explanation of the data preprocessing steps, description of the FS strategy, explanation of the filter, wrapper, and embedded methods of the FS algorithm, explanation of the FS classifiers employed, and discussion of the model performance metrics. Section 4 presents the results of the FS framework and provides metric estimates for the various classifier-based models utilized in classifying IBD patients from healthy controls. The outcomes of the study, including a comparative analysis of the performance against existing gene biomarker-based ML models, are discussed in Section 5. Finally, Section 6 concludes the research article by summarizing the main findings, highlighting limitations, and discussing potential future directions for further research. The ML-based framework, which screens potential gene biomarkers for classifying IBD samples from healthy control samples using gene microarray data, is visually represented in Figure 1.

## 2. Review of Literature

The literature review is organized into three main categories: (1) studies related to IBD in general, (2) studies specifically focused on UC, and (3) studies specifically focused on CD.

### 2.1. IBD (CD and UC)-Related Studies

Stemmer et al. [20] conducted a meta-analysis identifying 34 genes, including three novel long non-coding RNAs (lncRNAs), distinguishing inflamed IBD from non-IBD biopsies. They also found that 12 of 29 genes were upregulated in IBD blood, suggesting potential as non-invasive biomarkers. The study further explored potential therapeutic compounds for IBD using the Connectivity Map (CMap) database. Tang et al. [21] discovered *Ras homolog family member U* (RHOU) as a new IBD biomarker using support vector machine recursive feature elimination (SVM-RFE) and least absolute shrinkage selection operator (LASSO) regression methods. RHOU was validated through quantitative reverse transcription polymerase chain reaction (qRT-PCR) assays and receiver operating characteristic (ROC) analysis. The study also revealed RHOU correlations with immune cell populations. Yu et al. [22] recognized a 32-gene signature that accurately predicted IBD in an independent cohort with 86.5% accuracy using an XGBoost and uniform manifold approximation and projection (UMAP) techniques. Park et al. [23] developed a machine learning model using RNA sequencing data to distinguish inflammatory CD from UC with minimal error, identifying gene signatures that may help differentiate the two conditions. In 2019, Abbas et al. [24] proposed an integrative “Network-Based Biomarker Discovery (NBBD)” approach that combined network analysis and machine learning to identify a classifier with an AUC of 0.82 for distinguishing IBD patients from controls. Smolander et al. [25] compared the performance of support vector machines (SVMs) and deep belief networks (DBNs) in classifying breast cancer and IBD gene expression data. The study provided guidelines for effectively applying DBNs to complex genomics data classification. Biasci et al. [26] developed a 17-gene Quantitative Polymerase chain Reaction (qPCR)-based blood biomarker that could stratify IBD patients into high-risk and low-risk subgroups, predicting disease progression and treatment needs. Han et al. [27] presented a novel pathway-based approach called probabilistic pathway score (PROPS) that outperformed gene-based and alternative pathway-based classifiers in differentiating CD and UC. In 2017, Yuan et al. [28] used a two-step feature selection method and SVM to identify 21 gene biomarkers that could distinguish non-IBD from IBD samples with an accuracy of 0.937. In 2017, Isakov et al. [29] screened 347 potential gene biomarkers using an Elastic net method and built an IBD risk prediction model with high accuracy and AUC. In 2017, Chen et al. [30] used Bayesian hierarchical clustering on a large IBD cohort to develop a model that could predict IBD risk with AUC values of 0.70 for UC and 0.75 for CD. In 2015, Hubenthal et al. [31] used a penalized SVM method to identify a subset of 16 microRNAs from a pool of 863, which could distinguish individuals with and without disease with AUC values ranging from 0.89 to 0.98. In 2013, Wei et al. [32] implemented a two-step feature selection approach using the IIBCD dataset. They first applied a less strict association significance cutoff (<10^−4^) and minor allele frequency (>0.01) to filter genetic variants, then used LASSO (L1) penalization to screen 573 SNPs related to CD and 366 SNPs associated with UC. The resulting SVM-based model classified CD and UC patients from healthy controls with AUC values of 0.83 and 0.86, respectively.

### 2.2. Ulcerative Colitis (UC)-Related Studies

Qian et al. [33] identified five ferroptosis-related hub genes (*LCN2*, *MUC1*, *PARP8*, *PLIN2*, *TIMP1*) and built a high-performing logistic regression model to diagnose UC. Bu et al. [34] found four potential UC biomarkers (*HSPB3*, *ABCG2*, *VNN1*, *SLC6A14*) confirmed in an independent dataset (AUC = 0.889). They also observed immune cell differences, with UC having more γδ T cells, neutrophils, memory B cells, activated mast cells, and M1 macrophages. Zhang et al. [35] used machine learning methods to analyze microarray data from 387 UC patients and 139 healthy controls. They identified two genes, OLFM4 and C4BPB, that could effectively distinguish UC patients from controls with AUC > 0.8. These genes’ expression correlated with immune cell levels, suggesting involvement in UC pathogenesis. Khorasani et al. [36] developed an SVM model using a subset of 32 genes identified through feature selection. The model achieved high accuracy in detecting active UC and reasonable performance for inactive UC. Li et al. [37] used RF and artificial neural network approaches to develop a predictive model for UC diagnosis based on the expression of 30 differentially expressed genes. The model showed high predictive performance with an ROC-AUC of 0.95. Duttagupta et al. [38] explored circulating microRNAs in peripheral blood as non-invasive biomarkers for UC. They identified a signature of 31 differentially expressed, platelet-derived microRNAs that could distinguish UC patients from controls with 96.2% specificity, 89.5% sensitivity, and 92.8% accuracy.

### 2.3. Crohn’s Disease (CD)-Related Studies

Raimondi et al. [39] introduced a low-complexity neural network model for in silico CD diagnosis using whole exome sequencing data, outperforming previous approaches and providing interpretable insights. Romagnoni et al. [40] compared machine learning methods for classifying CD patients from controls using genotyping data, finding that non-linear models like gradient-boosted trees and neural networks can provide robust and complementary approaches. Wang et al. [41] developed an Analysis of Variation for Association with Disease (AVADx) method to predict Crohn’s disease (CD) status using exonic variants from genome/exome data. Their model, trained on 111 individuals, identified known CD genes and potential new ones. Bottigliengo et al. [42] investigated using Bayesian machine learning techniques, including Bayesian Network, Naive Bayes, and Bayesian Additive Regression Trees, to predict extra-intestinal manifestations in Crohn’s patients. However, the results showed poor performance compared to classical statistical tools. Daneshjou et al. [43] discussed the Critical Assessment of Genome Interpretation (CAGI) community experiment, which used CD Exomes sequencing data to predict phenotypes, highlighting such predictions’ challenges and potential applications. Pal et al. [44] utilized genotype data from the CAGI Crohn’s Exome challenge to train machine learning models that outperformed other approaches in predicting disease status. The resulting SVM-based model classified CD and UC patients from healthy controls with AUC values of 0.83 and 0.86, respectively. In 2013, Cui et al. [45] used Recursive SVM, a wrapper-based feature selection method, to identify 200 gene biomarkers. Leave-One-Out Cross-Validation (LOOCV) analysis demonstrated 88% accuracy, validated using an independent dataset.

This literature review highlights the diverse applications of machine learning in IBD research. The studies discussed demonstrate the potential of ML techniques to enhance our understanding of IBD pathogenesis and improve clinical management. Table 1 summarizes selected studies using gene selection and microarray datasets to identify diagnostic gene and microRNA biomarkers for IBD.

## 3. Materials and Method

This study employed a comprehensive approach to identifying and validating inflammatory bowel disease (IBD) gene biomarkers. We first described the datasets used for biomarker discovery and validation, followed by the data preprocessing steps. Differential gene expression analysis was conducted to identify significant genes differentially expressed between IBD and healthy control samples. Various feature selection methods were applied to select the most informative differentially expressed gene (DEG) biomarkers. We analyzed the expression patterns of these DEGs using Histogram Frequency Curve Plot (HFCP) analysis. The preprocessed microarray data was then split into training and testing sets, with the training set oversampled using SMOTE to address the class imbalance. Supervised machine learning models were trained using the selected DEG biomarkers, and their performance was evaluated using metrics such as accuracy and AUC-ROC. The validated DEG-based machine learning model was further tested on independent cohorts. Finally, gene ontology and pathway enrichment analyses were performed on the selected DEGs to gain insights into their role in IBD pathogenesis.

### 3.1. Dataset for Gene Biomarker Discovery and Validation

We used microarray data from the Gene Expression Omnibus (GEO) database to identify and validate IBD gene biomarkers. The GEO75214 cohort [17], consisting of 172 IBD patients and 22 healthy controls, was analyzed using the Affymetrix Human Gene 1.0 ST Array. This discovery cohort was used to identify differentially expressed gene (DEG) biomarkers with diagnostic potential for IBD. To validate the identified DEG biomarkers, we utilized two additional independent cohorts from GEO: GEO10616 [46] and GEO36807 [47]. We only considered the DEGs discovered in the original GEO75214 cohort during validation, excluding all other genes in the validation datasets.

### 3.2. Preprocessing Strategies for GEO75214 Key DEGs Dataset

We preprocessed the GEO75214 dataset using several techniques. Categorical variables were transformed using binary encoding, and quasi-constant features were removed. Outliers were detected and removed using the Interquartile Range method [48]. The data was then normalized using the Min–max algorithm to standardize the gene expression values [49]. These preprocessing steps ensured that the data was ready for further analysis.

### 3.3. Differential Gene Expression Analysis Methodology

We used an independent *t*-test to identify differentially expressed genes (DEGs) between IBD and control samples. The *t*-statistic and *p*-value were calculated for each gene, representing the significance of the difference in mean expression. We adjusted the *p*-values using the Benjamini–Hochberg method [50] to account for multiple tests. The fold change for each gene was calculated as the ratio of mean expression in IBD to control. The 95th percentile of the fold change distribution for non-DEGs was used as the fold change threshold. Genes were then categorized as upregulated, downregulated, or non-significant based on their adjusted *p*-value (*q*-values) and fold change. We created Venn diagrams, heatmaps, and volcano plots to visualize the DEGs. The volcano plot displayed the log2 fold change and negative log10 *p*-value, with genes colored by their category. The points on the plot are color-coded based on the gene category: “Upregulated” (red), “Downregulated” (blue), or “non-significant” (gray).

### 3.4. Feature Selection Approaches for Identification of Informative DEG Biomarkers

After identifying the DEGs, we applied several feature selection methods to select the most informative upregulated and downregulated biomarkers. These included filter-based (e.g., Mutual Information), wrapper-based (e.g., Recursive Feature Elimination), and embedded (e.g., Elastic Net, Gradient Boosting) approaches. We also used a feature complementation approach, selecting features unique to the up-and-down-regulated gene subsets identified using the different methods.

#### 3.4.1. Filter-Based Feature Selection

Filter-based feature selection is a technique for evaluating and ranking features based on their individual properties, such as correlation or mutual information with the target variable. It involves applying a statistical measure to each feature and selecting the top-ranked features for further analysis. Filter-based methods are computationally efficient and can handle high-dimensional datasets, making them popular for initial feature selection.

- Mutual Information Statistics [51]: Mutual information (MI) measures the mutual dependence between a gene’s expression (*X*) and the outcome (*Y*). The MI score is calculated as

(1) $$\mathrm{MI}\left(X,Y\right)=\sum \sum P\left(x,y\right)\times log\left(P\left(x,y\right)\;/\;\left(P\left(x\right)\times P\left(y\right)\right)\right)$$

Here, the notation *P*(*x*, *y*) denotes the joint probability distribution of the features *X* and *Y*.

The marginal probability distributions of the features *X* and *Y* are represented by *P*(*x*) and *P*(*y*), respectively.

Σ denotes the summation of all possible values of *X* and *Y*.

Mutual information measures the decrease in uncertainty about one variable (gene expression) when the value of the other variable (outcome) is known. Higher MI indicates a stronger association between the gene and outcome, suggesting biomarker potential. The scikit-learn parameters for mutual information feature selection are: (a) score_func = mutual_info_classif, (b) k = 2, (c) n_neighbors = 3, (d) random_state = None, (e) discrete_features = ‘auto’.

#### 3.4.2. Wrapper-Based Feature Selection

Wrapper methods evaluate feature subsets by training and testing a specific ML algorithm. They create different feature combinations, train models on each, and select the subset with the best performance on a predefined metric. Wrapper methods can capture complex feature interactions missed by filter methods.

- The Recursive Feature Elimination with Cross-Validation (RFECV) [52]: RFECV is a variation of Recursive Feature Elimination that automatically uses cross-validation to select the most informative genes for IBD classification. The scikit-learn parameters are:
  (a) estimator = ‘Randomforestclassifier’, (b) step = 1, (c) min_features_to_select = 10, (d) cv = 5, (e) scoring = ‘roc_auc’.

#### 3.4.3. Embedded Feature Selection

Embedded methods integrate feature selection into the learning algorithm itself. They aim to identify the most relevant features during model training by incorporating feature selection as a step within the algorithm. Embedded methods are well-suited for high-dimensional datasets.

- Elastic Net [53]: Elastic Net is a regularization technique combining L1 (Lasso) and L2 (Ridge) penalties to select features. It shrinks some feature weights to zero, effectively excluding those features. This allows Elastic Net to select groups of highly correlated features, making it effective for high-dimensional, correlated data. The Elastic Net parameters in scikit-learn are as follows: alpha: 1.0, max_iter: 1000, fit_intercept: True, $l1_{ratio}$: 1.0, normalize: False, max_features = 2, tol: 1e−4. The Elastic Net objective function is:

Minimize:

(2) $$\left(1∕\left(2\times n_{samples}\right)\right)\times {\left\|y-Xw\right\|}^{2}+\alpha \times \left(l1_{ratio}\times {\left\|w\right\|}_{1}+0.5\times \left(1-l1_{ratio}\times {\left\|w\right\|}_{2}^{2}\right)\right)$$

In the above equation,

*y* represents the target variable.

*X* is the feature matrix.

*w* is the weight vector that indicates the importance of each feature.

$n_{samples}$ is the number of samples in the dataset.

*α* is a hyperparameter that controls the regularization strength.

$l1_{ratio}$ is a hyperparameter determining the balance between the L1 and L2 penalties.

The Elastic Net objective function consists of two components:

The squared loss term ${\left\|y-Xw\right\|}^{2}$, which measures the deviation between the predicted values and the actual target values.

The regularization term, which consists of two parts:

The L1 penalty ${\left\|w\right\|}_{1}$ encourages sparsity in the weight vector, leading to feature selection.

The L2 penalty ${\left\|w\right\|}_{2}^{2}$ encourages small weights, preventing overfitting.

- Gradient Boosting Classifier Feature Selection: This method uses a gradient-boosting classifier to assess feature importance for classification tasks. Feature importance is determined by measuring the reduction in impurity from splits on each feature during decision tree construction in gradient boosting. The most important features can be identified based on their importance scores [54]. The Gradient Boosting Classifier parameters in scikit-learn are as follows: estimator: GradientBoostingClassifier (), max_features: 2, number of estimators: 100, min_samples_leaf: 1, learning_rate: 0.1, max_depth: 3, min_samples_split: 2, subsample: 1.0. The mathematical equation for feature importance in a gradient-boosting classifier can be expressed as follows:

Importance (feature) = ∑ (gain in impurity due to splits on the feature)/(total gain in impurity)(3)

In this equation:

- “Importance (feature)” represents the importance score of a specific feature.
- “Gain in impurity due to splits on the feature” refers to the reduction in impurity achieved by splitting on that feature during the construction of decision trees in the gradient boosting process.
- “Total gain in impurity” represents the total reduction in impurity across all features.

### 3.5. Histogram Frequency Curve Plot (HFCP) Analysis

The HFCP visualizes the distribution of gene expression levels (continuous features) between IBD patients and healthy controls in the GEO75214 dataset. It highlights differences in mean gene expression between the two groups.

### 3.6. Partitioning of Transformed Microarray Dataset

The dataset was split into 65% training and 35% test sets. The training set had 126 samples (111 IBD, 15 healthy), while the test set had 69 samples (61 IBD, eight healthy).

### 3.7. Oversampling of Training Data Using SMOTE

To address the class imbalance, the minority class (healthy) in the training set was oversampled using the Synthetic Minority Oversampling Technique (SMOTE) [55]. This generated synthetic samples to balance the class distribution, but the test set remained unchanged. The SMOTE parameters used in scikit-learn are as follows: (a) sampling_strategy = ‘auto’, (b) random_state = 3, and (c) k_neighbors = 3.

### 3.8. Leave-One-Out Cross-Validation (LOOCV)

LOOCV [56] was used to assess model performance on the training set. Each data point served as the validation set, and the aggregated confusion matrix was used to compute the average accuracy.

### 3.9. Training of Supervised Learning Models

The selected gene biomarkers were trained to train supervised learning classifiers, including Logistic Regression, K-Nearest Neighbors, Gaussian Naive Bayes, Support Vector Classifier, Random Forest, Multi-Layer Perceptron, and Decision Tree.

#### Supervised Learning Classifiers

- Logistic Regression [57]: LR computes the probability of a sample being assigned to a specific class. The probability is obtained using the logistic (sigmoid) function. The coefficients (w0, w1, w2, …, wn) are estimated during the training process. The parameters used for the LR classifier in scikit-learn are as follows: fit_intercept = True, penalty = “l2”, dual = False, intercept_scaling = 1, C = 1, tol = 0.0001, multi_class = “auto”, class_weight = None, verbose = 0, max_iter = 100, solver = “liblinear”, warm_start = False, random_state = 123. The LR equation for classifying IBD and healthy control samples is as follows:

(4) $$P\left(y=1|x\right)=1/\left(1+exp^{\left(-z\right)}\right)$$

where *z* is the linear combination of the selected gene biomarkers and their corresponding coefficients:

(5) z=w0+w1x1+w2x2+⋯+wn×xn

- Support Vector Classifier (SVC): The SVC identifies the most favorable hyperplane that effectively differentiates the classes within the input domain. The decision function is defined as

(6) $$f\left(x\right)=\mathrm{sign}\left(w\cdot \phi \left(x\right)+b\right)$$

In the equation, the weight vector is denoted as w, ϕ(x) represents the feature transformation (such as mapping gene biomarkers to a higher-dimensional space using kernel functions), and b represents the bias term.

- Decision Tree (DT) [58]: DTs generate predictions using a hierarchical structure of decision and leaf nodes. At each decision node, a selected gene biomarker is compared to a threshold value and the prediction is made by traversing the tree structure based on the feature values. In binary classification, the classes are usually denoted as “0” and “1”. The DT algorithm calculates the Gini index for each potential split. The Gini index can be calculated using the following equation:

(7) $$Gini\;Index=1-\left(p_{0}^{2}+p_{1}^{2}\right)$$

Here, 

the symbol $p_{0}$ denotes the probability associated with an instance in the class labeled as “0”.

$p_{1}$ represents the probability of an instance being assigned to class “1”.

The parameters used for the DT classifier in scikit-learn are as follows: max_depth = 7, criterion = “gini,” random_state = 1, min_samples_split = 3, min_impurity_decrease = 0, splitter = “best,” max_features = None, min_samples_leaf = 1, max_leaf_nodes = None, class_weight = None, alpha = 0, min_weight_fraction_leaf = 0.

- Random Forest (RF) [59]: RF combines multiple DTs. The final prediction aggregates the predictions from each tree, e.g., by majority voting. The RF parameters are random_state = 123, n_estimators = 1000, and max_depth = 5.
- Gaussian Naïve Bayes (GNB) [60]: GNB assumes the gene biomarkers follow a Gaussian distribution. The class probability is calculated using Bayes’ theorem and feature independence. The GNB parameters in scikit-learn are as follows: priors = None, var_smoothing = 1 × 10^−9^. For binary classification, the equation simplifies to a ratio of probabilities:

(8) $$P\left(y|x_{1},x_{2},\ldots ,x_{n}\right)=\left(P\left(y\right)\times ⊓P\left(x_{i}|y\right)\right)∕P\left(x_{1},x_{2},\ldots ,x_{n}\right)\;$$

Here, 

the posterior probability of class ‘*y*’, given the feature values $x_{1},x_{2},\ldots ,x_{n}$ is represented as $P\left(y|x_{1},x_{2},\ldots ,x_{n}\right)$.

*P*(*y*) is the prior probability of class “*y*”.

$P\left(x_{i}|y\right)$ is the likelihood of feature $x_{i}$ given class “*y*”.

$P\left(x_{1},x_{2},\ldots ,x_{n}\right)$ is the probability of observing the feature values $x_{1},x_{2},\ldots ,x_{n}$.

eXtreme Gradient Boosting Classifier (XGBoost) [61]: XGBoost optimizes weak model weights to minimize a loss function using gradient descent and regularization. It offers advanced features like custom loss functions and handling missing values. The mathematical equation for XGBoost can be represented as

(9) $$y_{hat}=\sum \left(w\times h\left(x\right)\right)$$

In the given equation, 

$y_{hat}$ represents the predicted outcome.

∑ denotes the summation.

w represents the weight associated with each model in the ensemble.

$h\left(x\right)$ represents the prediction made by each weak model (e.g., decision tree) in the ensemble.

The parameters used for the XGBoost classifier in scikit-learn are as follows: n_estimators: 100, max_depth: 3, subsample: 1.0, colsample_bytree: 1.0, reg_alpha: 0.0, reg_lambda: 1.0, min_child_weight: 1, gamma: 0.0, random_state: None, learning_rate: 0.1.

- Multi-Layer Perceptron (MLP) [62]: MLP involves forwarding gene biomarkers through interconnected neurons and calculating outputs using activation functions. The MLP parameters used for the XGBoost classifier in scikit-learn are as follows: hidden_layer_sizes = 100, activation = “relu”, solver = “adam”, alpha = 0.0001, shuffle = True, learning_rate_init = 0.001, learning_rate = “constant”, max_iter = 200, power_t = 0.5, tol = 0.0001, batch_size = “auto”, nesterovs_momentum = True, warm_start = False, early_stopping = False, verbose = False, beta_1 = 0.9, n_iter_no_change = 10, validation_fraction = 0.2, epsilon = 1 × 10^−8^, beta_2 = 0.999, max_fun = 15,000, random_state: = 123, momentum = 0.9.

### 3.10. Evaluating Model Performance

We assessed the diagnostic classifier’s performance using standard metrics like the confusion matrix, accuracy, and the AUC-ROC curve [56].

### 3.11. Validating the DEGs-Based ML Model

We used two independent cohorts, GEO10616 and GEO36807, to validate the gene biomarkers identified from the discovery cohort (GE75214). This allowed us to assess the reliability and applicability of the selected biomarkers.

### 3.12. Pathway Analysis of Selected DEGs

We conducted Gene Ontology (GO) and Kyoto Encyclopedia of Genes and Genomes (KEGG) pathway enrichment analysis on the six identified DEGs (*VWF*, *IL1RL1*, *DENND2B*, *MMP14*, *NAAA*, and *PANK1*) using the ORA method in WebGestalt 2024 [19]. We identified significantly enriched terms using a *p*-value cutoff of 0.05.

## 4. Results

The results section presents the key findings of this study in a structured manner. We begin with an overview of the identified differentially expressed genes (DEGs) from the microarray dataset, including up and downregulated DEGs. Visualizations such as heat maps, volcano plots, and Venn diagrams are then used to enhance understanding of gene expression patterns. We then describe the feature selection approach employed to filter the DEGs and the statistical analysis to identify the most significant gene biomarkers. The performance of various supervised machine learning models using these gene biomarkers is evaluated, and the optimal Random Forest (RF) model is selected and tuned. The generalizability and robustness of the RF-based model are then demonstrated through independent validation on external cohorts, and its superior performance is highlighted compared to other published models. Finally, we present the gene ontology and pathway enrichment analysis of the key upregulated and downregulated DEGs to gain insights into the underlying biological processes and mechanisms associated with inflammatory bowel disease.

### 4.1. Identification of DEGs from GSE75214

We obtained gene expression dataset GSE75214 with microarray data from IBD and normal samples. The GPL6244 platform, Affymetrix Human Gene 1.0 ST Array, was used. Comparative analysis identified 2239 significant DEGs between IBD and Normal groups from the GSE75214 cohort.

### 4.2. Identification of Top DEGs in IBD vs. Control

Comparative analysis identified the top 10 upregulated genes: *DENND2B*, *LCN2*, *IFITM3*, *SLC6A14*, *BACE2*, *S100A11*, *PLS3*, *PARP8*, *NFKBIZ*, *DUOX2*, ranked by *p*-value. The top 10 downregulated genes were: *CNTFR*, *RNY1P5*, *STBD1*, *HINFP*, *PAQR5*, *CNTN4*, *RNF135*, *FRMD1*, *SFXN1*, *SLC38A4*, also ranked by *p*-value. See Table 2 for the top 10 upregulated and downregulated genes and Supplementary Tables S1 and S2 for the comprehensive lists.

### 4.3. Visualizing Upregulated and Downregulated DEGs from GSE75214

**Figure 2:**
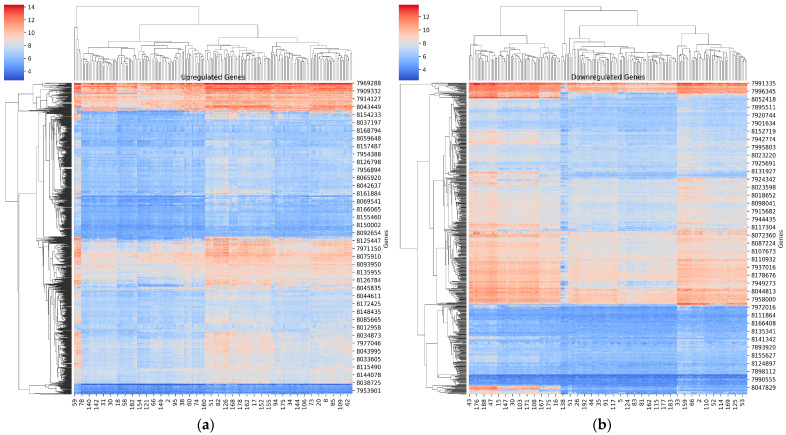
Differential Gene Expression Patterns between IBD and Normal samples of the GSE75214 cohort. (**a**) The Figure displays the heatmap results of the upregulated genes between the IBD and Normal subjects. (**b**) The Figure displays the heatmap results of the downregulated genes between the IBD and Normal subjects. The color scale ranges from dark blue, indicating low expression, to dark red, indicating high expression. The expression levels provide insights into the contrasting gene expression patterns associated with IBD and Normal subjects.

Figure 2a shows a heatmap of the top three upregulated genes in each main cluster. Cluster 1 has *SLC6A14*, *DUOX2*, *MMP3*; Cluster 2 has *DUOXA2*, *MMP1*, *LCN2*; Cluster 3 has *IDO1*, *S100A8*, *SAA2*, *IL1B*. Figure 2b shows a heatmap of each main cluster’s top three downregulated genes. Cluster 1 has *PRKG2*, *MT1M*, *SLC26A2*; Cluster 2 has *SLC13A1*, *HMGCS2*, *UGT2A3*; Cluster 3 has *CYP2B6*, *ABCG2*, *TMIGD1*, *MEP1B*.

**Figure 3:**
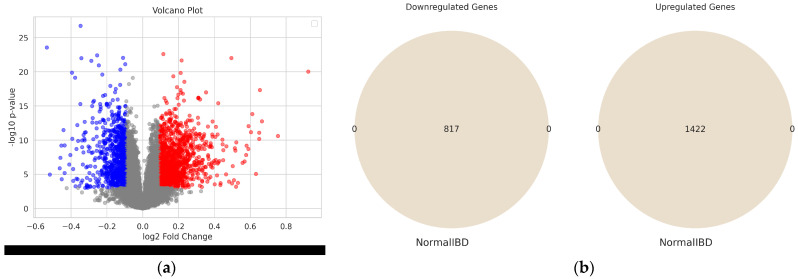
Analysis of DEGs between IBD and Healthy Controls from the GSE75214 cohort. (**a**) The volcano plot illustrates the DEGs observed between IBD and normal individuals in the GSE75214 cohort. The y-axis represents the negative logarithm (base 10) of the *p*-value, while the x-axis represents the log2 fold change. The significant DEGs, meeting the criteria of a *p*-value less than 0.001 and a fold change exceeding the threshold of 1.06712, are highlighted on the plot. (**b**) Venn diagram illustrating the overlap of DEGs in the GSE75214 cohorts. The diagram shows the genes that are common DEGs (upregulated and downregulated) between the two groups (IBD and Normal) of the GSE75214 cohort.

The Volcano plot in Figure 3a visualizes the statistically significant DEGs based on *p*-value < 0.001 and fold change > 1.06712 for the GSE75214 dataset. The Venn diagram in Figure 3b shows the overlap of DEGs between IBD and control groups. There were 1422 overlapping upregulated genes and 817 overlapping downregulated genes shared between the two groups. No unique DEGs were detected.

### 4.4. Feature Selection for Potential IBD Biomarkers

A feature selection (FS) approach was used to identify potential IBD biomarkers from the DEGs in the GSE75214 dataset. Four different FS techniques were applied, and the unique features from each method were combined into a master feature subset (Table 3).

A master feature subset was created by selecting and combining the unique features from the subsets of features obtained from the four different FS methods. Consequently, a master feature subset consisting of six gene biomarkers was generated, as shown in Table 4.

### 4.5. Results of Two-Tailed Unpaired T-Test on Potential IBD Biomarkers

A two-tailed unpaired *t*-test with a significance level of 5% was performed to identify which of the six selected gene biomarkers showed significant differences in mean expression between the IBD and healthy control groups. The results of this *t*-test analysis are presented in Table 5. All six gene biomarkers (*VWF*, *IL1RL1*, *DENND2B*, *MMP14*, *NAAA*, and *PANK1*) had *p*-values less than 0.05, indicating that their mean expression levels significantly differed between the IBD and control samples.

### 4.6. Potential IBD Biomarkers Identified

**Figure 4:**
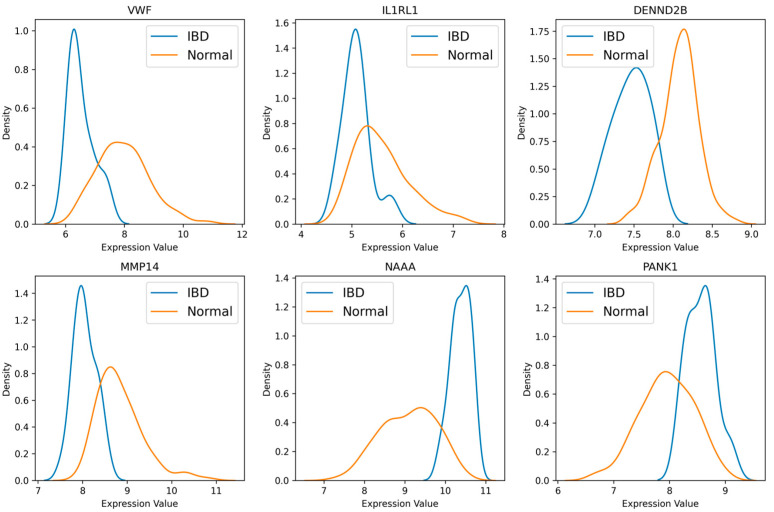
KDE subplots illustrate the expression distribution of six genes (*VMF*, *IL1RL1*, *DENND2B*, *MMP14*, *NAAA*, and *PANK1*) across two groups (IBD patients and Normal controls).

All six gene biomarkers (*VWF*, *IL1RL1*, *DENND2B*, *MMP14*, *NAAA*, and *PANK1*) showed significant differences in mean expression levels between the IBD and healthy control groups, as confirmed by the two-tailed unpaired *t*-test (*p* < 0.05). The frequency distribution plots in Figure 4 further illustrate the significant differential expression of these six DEG biomarkers across the IBD and control samples. Based on these findings, the final set of six gene biomarkers (*VWF*, *IL1RL1*, *DENND2B*, *MMP14*, *NAAA*, and *PANK1*) will be used to build and evaluate the best-supervised classification model to distinguish IBD patients from healthy controls.

### 4.7. Screening the Best-Performing Classification Model

**Figure 5:**
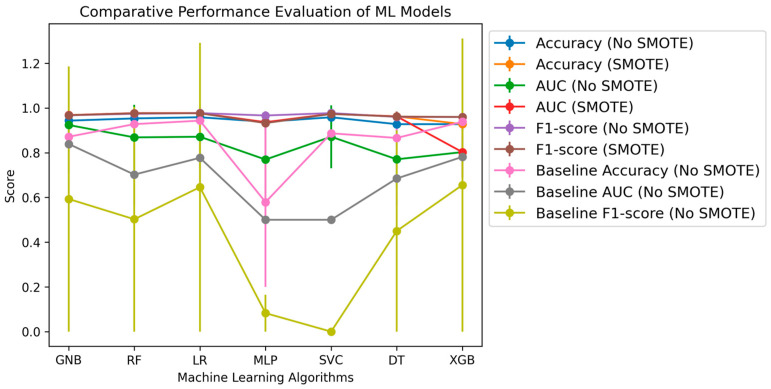
Comparison of Accuracy, F1-Score, and AUC Scores between ‘Six Gene Biomarkers’ and ‘Baseline (33,253 Genes)’ based ML models with SMOTE and without SMOTE. Error bars represent the standard deviation values for each performance evaluator.

The study aimed to identify the most effective classification model for distinguishing IBD from healthy control samples using the set of six potential DEG biomarkers identified earlier. As shown in Figure 5, the supervised classification models were evaluated using the six biomarker features and a baseline set of 33,253 gene features.

The results indicate that the Random Forest (RF) model outperformed the other supervised learning algorithms when utilizing the six selected biomarker features. Based on leave-one-out cross-validation, the RF model achieved the highest aggregated F1 score (0.97628 ± 0.0150), accuracy (0.9767 ± 0.0148), and AUC (0.9767 ± 0.0148) (Table 6). These findings suggest that using the RF classification model, the six DEG biomarkers (*VWF*, *IL1RL1*, *DENND2B*, *MMP14*, *NAAA*, and *PANK1*) can effectively distinguish IBD patients from healthy controls. This approach holds promise for earlier, more accurate diagnosis of IBD.

### 4.8. Tuning and Validating the Random Forest Classifier

The optimal hyperparameters for the Random Forest (RF) classifier were determined using the GSE75214 training dataset containing the 6 gene biomarkers: n_estimators: 200, max_depth: None, max_features: ‘sqrt’, min_samples_split: 5, and min_samples_leaf: 2. To validate these hyperparameters, 5-fold cross-validation was performed, yielding: F1 Score: 0.9870 ± 0.013, Accuracy: 0.9855 ± 0.0145, and AUC: 0.992 ± 0.018.

**Figure 6:**
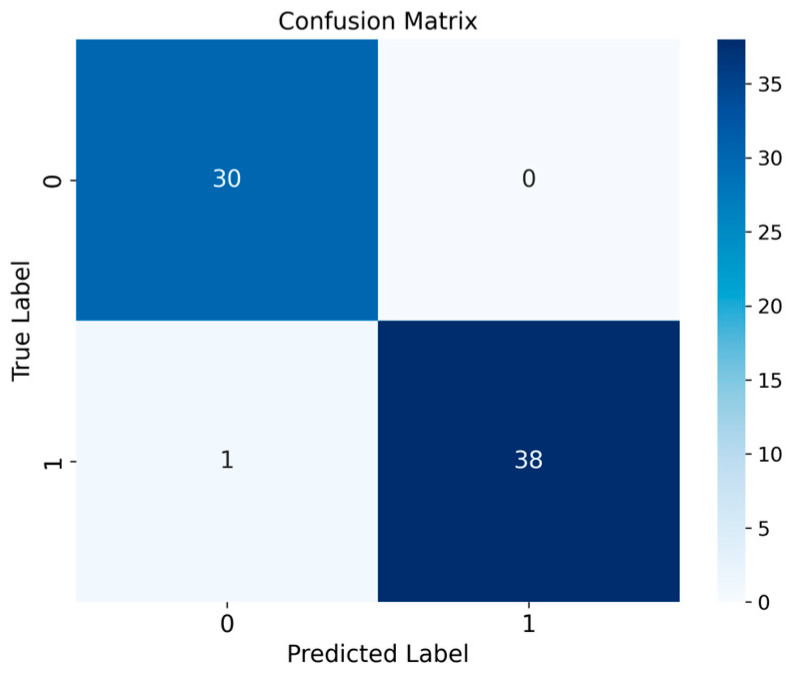
Illustrates a visualization of the optimized RF-based classification model’s performance using a confusion matrix.

The consolidated confusion matrix in Figure 6 illustrates the optimized RF model’s performance on independent test data. In this matrix, the positive class represents IBD, and the negative class represents healthy controls. The validation process improved the classification of true positives and negatives, as evidenced by the higher average accuracy, F1-score, and AUC values.

### 4.9. Evaluating Model Generalizability and Robustness

**Figure 7:**
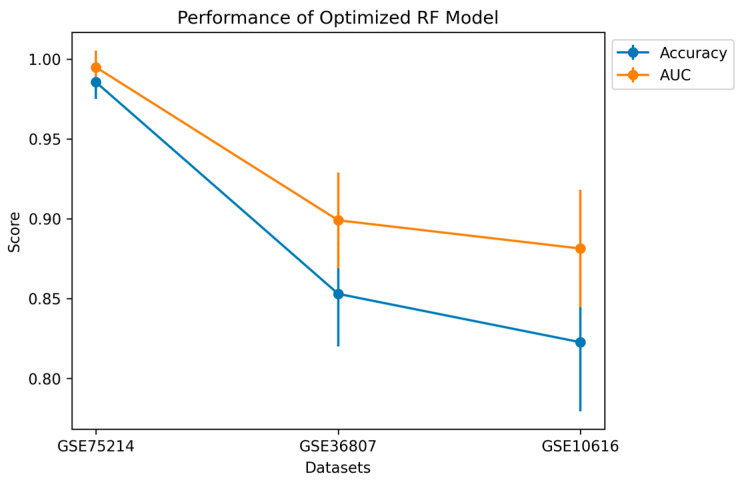
Performance of six biomarker-based optimized RF models on different IBD cohorts (GDE30687 and GSE10616).

The optimized RF model, trained and tested using the 6-gene biomarker dataset, was further evaluated for generalizability and robustness across different IBD gene expression cohorts. As shown in Figure 7, the RF model exhibited strong performance on the GSE10616 cohort (Accuracy: 0.820 [CI: 0.806–0.834], AUC: 0.880 [CI: 0.870–0.890]) and the GSE36807 cohort (Accuracy: 0.850 [CI: 0.842–0.858], AUC: 0.900 [CI: 0.895–0.905]). These results confirm the RF model’s ability to effectively adapt to the GSE36807 and GSE10616 datasets, demonstrating its potential for accurately classifying IBD versus healthy individuals across different cohorts.

### 4.10. Comparative Performance Evaluation

**Figure 8:**
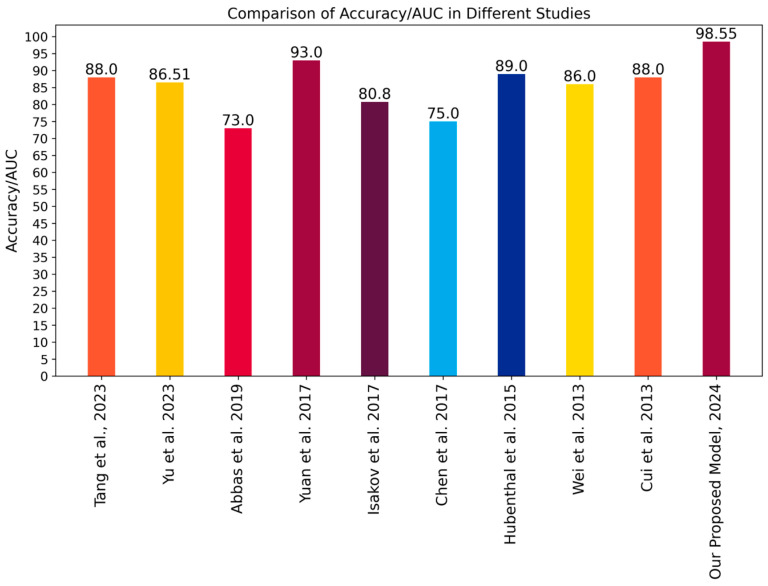
Presents a comparative evaluation of accuracy and AUC values between our and related models [21,22,24,28,29,30,31,32,45].

Figure 8 compares the performance of our proposed 6-gene biomarker-based RF classification model against other published models. Our model achieved an accuracy of 0.9855 ± 0.0145 and an AUC of 0.992 ± 0.018, outperforming the other gene biomarker-based models. These results indicate that the 6-gene biomarker-based RF model has superior classification capability compared to previous approaches. This suggests that the 6-gene signature could significantly contribute to earlier IBD diagnosis, improved treatment strategies, and more personalized patient management.

### 4.11. Gene Ontology and Pathway Enrichment Analysis of the Six Key DEGs

The Overrepresentation analysis (ORA) method, available in the WebGestalt developed by Wang et al. in 2017, was utilized to perform enrichment analysis of GO and KEGG pathways for the six DEGs (*VWF*, *IL1RL1*, *DENND2B*, *MMP14*, *NAAA*, and *PANK1*). The significance cutoff value of *p* < 0.05 was employed to determine enriched terms. The analysis of GO biological pathways revealed that the upregulated genes (*VWF*, *IL1RL1*, *DENND2B*, *MMP14*) were associated with several biological processes, including cell-substrate adhesion, positive regulation of cell activation, extracellular structure organization, regulation of leukocyte activation, and interleukin-5 production. In addition, the analysis of KEGG and reactome pathways revealed significant enrichment of the upregulated genes in pathways such as GnRH signaling pathway, platelet activation, complement and coagulation cascades, ECM-receptor interaction, parathyroid hormone synthesis, secretion and action, TNF signaling pathway, Integrin signaling, and extracellular matrix organization, as presented in Table 7.

The two downregulated DEGs, *NAAA* and *PANK1*, were linked to processes like ribose phosphate biosynthesis, cofactor biosynthesis, nucleoside metabolism, neurotransmitter transport, purine biosynthesis, neurotransmitter regulation, nucleoside phosphate biosynthesis, and coenzyme metabolism. Pathway analysis also showed these genes were enriched in pantothenate/CoA biosynthesis, neurotransmitter release, vitamin/cofactor metabolism, and chemical synaptic transmission pathways, as shown in Table 8. These findings provide insights into the biological processes and pathways affected by the DEGs.

## 5. Discussion

IBD is a chronic inflammatory disorder characterized by persistent symptoms and relatively low mortality. However, the increasing global prevalence of IBD has strained healthcare systems. While the precise cause of IBD is uncertain, understanding the disease’s pathology and molecular mechanisms is crucial for improving diagnosis and treatment. We can identify potential diagnostic biomarkers by leveraging gene expression data and bioinformatics/ML analysis. This study aimed to use the GSE75214 dataset as the primary cohort, with GSE36807 and GSE10616 as validation cohorts. We identified 1422 upregulated and 817 downregulated differentially expressed genes (DEGs) in GSE75214. Our analysis uncovered six potential gene biomarkers (*VWF*, *IL1RL1*, *DENND2B*, *MMP14*, *NAAA*, and *PANK1*) with strong diagnostic potential. Notably, DENND2B and PANK1 represent novel IBD biomarkers. Integrating these six genes into a Random Forest model achieved exceptional performance, with an AUC of 0.992 ± 0.018 and an accuracy of 0.9855 ± 0.0145. Validation on independent cohorts confirmed the model’s robustness. Our study provides novel insights into IBD-associated genes, introduces an innovative ML approach, and highlights *DENND2B* and *PANK1* as new biomarker candidates. These findings could significantly impact IBD research and diagnostics.

Our gene ontology and pathway analysis enhanced our understanding of the processes involved in IBD. The analysis revealed that the upregulated genes, namely *IL1RL1*, *MMP14*, and *VWF*, are involved in key cellular processes. Thus, our upregulation profiling of *IL1RL1* corroborates with earlier studies showing that *IL1RL1* is upregulated in IBD patients. In the context of IBD, *IL1RL1*, through its product ST2, may contribute to regulating immune responses in the gut. This finding is significant as *IL1RL1* exhibits preferential expression on colonic T-regulatory cells, supporting their function and adaptation to the inflammatory environment. This is crucial in preserving gut homeostasis and potentially attenuating the excessive inflammation associated with IBD [63,64].

*MMP14*, a matrix metalloproteinase, participates in extracellular matrix degradation, which is essential for tissue remodeling and healing. In IBD, excessive MMP activity and insufficient tissue inhibitors of metalloproteinases (TIMPs) inhibition can contribute to mucosal damage and inflammation [65,66,67]. Our findings corroborate previous studies showing *MMP14* upregulation in IBD patients.

*VWF* is implicated in blood coagulation, platelet adhesion, and wound healing. Elevated *VWF* levels in active IBD may stem from vascular injury or inflammatory mediator release and contribute to the increased thrombosis risk [68,69]. Monitoring *VWF* can assist in IBD hemostasis management [70]. Importantly, our findings also show that *VWF* is upregulated in IBD patients compared to normal samples, further signifying the importance of this gene in our study.

The *DENND2B* gene, with its predicted guanyl-nucleotide exchange factor activity, could potentially influence MAPK signaling pathways [71,72,73]. *DENND2B*’s activation of Rab13 enhances the invasive potential of epithelial cancers [74,75]. Conversely, disrupting this *DENND2B-Rab13* signaling axis significantly impairs the spread and migratory capacity of highly aggressive epithelial cancer cells in vitro and in vivo [76,77]. Our data revealed *DENND2B* overexpression in IBD, hinting at its role in inflammation and healing, although its specific function requires further research. These findings open possibilities for therapeutic interventions targeting *DENND2B* in IBD and cancer.

The gene ontology and enrichment analysis show that the downregulated genes, *NAAA* and *PANK1*, are involved in pantothenate/CoA biosynthesis, neurotransmitter regulation, and transport (Table 8). *NAAA* has limited reported connections to IBD, but studies found decreased *PPAR*, *PPAR*, and *NAAA*, with increased *FAAH* and *iNOS*, in colitis mucosa [78]. Another study identified *NAAA* as a potential UC biomarker [79]. *NAAA* modulates the endocannabinoid system, which is altered in IBD and influences inflammation and pain [80,81,82]. Therefore, our current study findings suggest that decreased levels of *NAAA* expression may alter the endocannabinoid signaling pathway, thereby affecting endocannabinoid molecules’ anti-inflammatory effect, leading to inflammation and pain in IBD patients.

*PANK1* codes the rate-limiting enzyme in CoA synthesis from pantothenate [83]. *PANK1* is associated with CoA biosynthesis, phosphorylation, and acetyl-CoA regulation. Altered CoA metabolism can affect gut epithelium energy and inflammation in IBD [84]. Moreover, the decreased *PANK1* level observed in our gene expression analysis in IBD patients suggests intracellular CoA changes may impact the gut epithelium. Research also reveals *PANK1*’s potential in cancer. *PANK1* can inhibit hepatocellular carcinoma by regulating Wnt/β-catenin [85] and modulating the cell cycle [86,87]. Bioinformatic analysis identified *PANK1* as differentially expressed between normal and tumor tissues [87]. *PANK1* expression correlates with prognosis, tumor immunity, and metabolism in renal cell carcinoma [88]. These findings suggest *PANK1*’s importance as a therapeutic target and prognostic biomarker in various cancers.

The identified upregulated and downregulated DEGs have significant roles in IBD and cancer, warranting further research on their therapeutic implications. However, the study has limitations that require consideration. The primary findings need validation in larger clinical cohorts. Additionally, immune cell infiltration studies are essential to assess composition and correlations with IBD pathogenesis. Such investigations may yield new insights into the molecular mechanisms underlying IBD.

## 6. Conclusions

In conclusion, our research identified a six-gene signature, including novel biomarkers *DENND2B* and *PANK1*, that effectively distinguished active IBD from healthy controls. Functional analysis revealed the signature genes were associated with key pathways in IBD pathogenesis, such as complement/coagulation, neurotransmitter regulation, and CoA biosynthesis. This six-gene signature demonstrated diagnostic potential beyond IBD, highlighting its versatility.

Future priorities include molecular validation of the biomarkers using qRT-PCR and investigating immune cell infiltration to provide deeper insights into IBD pathogenesis. Overall, our integrative approach of transcriptomics, machine learning, and high-throughput technologies advances the understanding and management of complex diseases like IBD. These findings lay the foundation for further research into genetic biomarkers with diagnostic and therapeutic implications.

## Figures and Tables

**Table 1:** Comparison of various ML-based studies for selecting a subset of potential biomarkers to classify IBD patients from healthy control samples.

Studies	Feature Selection Methods	Machine Learning Algorithm (s)	Modality	IBD Type	Performance Measures [Accuracy/AUC]	Outcome	Limitations
IBD (UC/CD) Related Studies							
Stemmer, 2024 [20]	F-test in one-way analysis of variance (ANOVA F-test) value	K-nearest neighbor (KNN), Naïve Bayes, Extra Trees, and RF	Gene Expression Profiles	UC/CD	Best model AUC: 0.95	Diagnosis of IBD	Limited validation cohort. Further validation is required.
Tang, 2023 [21]	SVM-RFE and LASSO regression	SVM	Gene Expression Dataset	CD/UC	SVM accuracy: 0.8	Diagnosis of IBD	Incomplete clinical information. Limited validation cohort. Further validation is required. Need for more personalized studies.
Yu, 2023 [22]	XGBoost and UMAP	XGBoost	Expression data (microarray and RNA-seq)	UC/CD	XGBoost accuracy: 0.865	Diagnosis of IBD	More features increase complexity and training time. No parameter tuning for the XGBoost algorithm
Park, 2021 [23]	Sparse partial least-squares discriminant analysis	Partial Least-Squares Discriminant Analysis (PLS-DA)	RNA-seq analysis	UC/CD	Average error rate across classes: 0.155	Classification of UC and CD	Not validated in an independent cohort.
Abbas, 2019 [24]	The RF feature importance score and NBBD scores	RF classifiers	Large pediatric IBD metagenomics dataset	Peds CD/UC	RF: Accuracy 0.73, AUC 0.82		Lack of biological significance evaluation for identified interactions. No validation cohort.
Smolander, 2019 [25]	DBNs and SVM	DBNs and SVM	Gene Expression datasets	CD/UC	DBNs: UC accuracy 0.9706, CD accuracy 0.9703	Diagnosis of IBD	Complexity of the models
Biasci, 2019 [26]	Logistic Regression (LR) with an adaptive Elastic-Net penalty	qPCR classifier	Gene Expression Profiling	CD/UC	17-gene qPCR classifier: - High sensitivity: 72.7% CD, 100% UC - High negative predictive value: 90.9% CD, 100% UC - Hazard ratios: 2.65 CD, 3.12 UC.	Diagnosis of IBD	Non-interventional, real-world design. Unclear performance in patients on induction therapy. An interventional study is needed to confirm clinical utility.
Han, 2018 [27]	-	RF, LR, and conditionally responsive genes (CORG)	Gene Expression dataset	CD/UC	Gene-based feature sets had a validation AUC range of 0.6 to 0.76	Diagnosis of IBD	Limited validation cohort. Further validation is required.
Yuan, 2017 [28]	Minimum Redundancy and Maximum Relevance (mRMR) Incremental Feature Selection (IFS) Shortest-Path (SP)	Sequential minimal optimization	Gene Expression datasets	CD/UC	Highest accuracy: 0.9370 with 21 genes	Risk of IBD	Predictive accuracy of 20-gene biomarkers not estimated. The classification task was computationally expensive. The classification task had limited interpretability.
Isakov, 2017 [29]	Elastic net regularized generalized linear model.	Combined model involving RF, SVM, gradient boosting, and elastic net	Expression data (microarray and RNA-seq)	CD/UC	Combined model: Accuracy: 0.808 AUC: 0.829	Risk of IBD	Complex decision-making due to increased features.
Chen, 2017 [30]	Bayesian mixture approach	Polygenic score, elastic-net regularization, best linear genomic prediction, and a Bayesian mixture mode	GWAS or Immunochip SNP data	CD/UC	CD AUC: 0.75, UC AUC: 0.70	Risk of IBD	Broader variant inclusion did not improve risk prediction.
Hübenthal, 2015 [31]	Penalized SVM	RF	MicroRNAs	CD/UC	17-gene classifier holdout AUC: 0.75 to 1.00 (including indeterminate) 0.89 to 0.98 (excluding indeterminate)	Diagnosis of IBD	Small sample sizes. Further evaluation in larger, independent cohorts is needed. Cohorts should have well-defined clinical characteristics.
Wei, 2013 [32]	Lasso penalization, relaxed significance cutoff (<10^−4^)	SVM, gradient boosted tree	Genetics, Immunochip	CD/UC	UC AUC 0.83, CD AUC 0.86	Risk of IBD	Increased feature screening complexity leads to complex decision-making. No SVM parameter tuning. No gradient-boosted tree parameter tuning.
Ulcerative Colitis (UC)-Related Studies							
Qian, 2023 [33]	LASSO	Naïve Bayes, Logistic, IBk, Random Forest	Gene Expression datasets	UC	LR model: AUC-Training: 1.000, Validation: 0.995	Diagnosis of UC	No experimental validation. A larger sample size is required. Need laboratory validation.
Bu, 2022 [34]	LASSO regression model and SVM-RFE	LASSO regression model and SVM-RFE algorithm.	Gene Expression Profiles	UC	4-gene model AUC: 0.977	Diagnosis of UC	Limited data requires external validation. Need prospective studies to evaluate biomarker utility.
Zhang, 2022 [35]	RF, SVM-RFE, Principal component analysis (PCA), Gradient Boosting Machine (GBM) and LASSO regression	RF, SVM, PCA, GBM and LASSO regression	Gene Expression Profiles	UC	SVM model AUC: 0.915	Diagnosis of UC	Insufficient verification
Khorasani, 2020 [36]	Dimension reduction through perturbation theory (DRPT)	SVM	Gene Expression dataset	UC	Active UC AUPRC: 1.0 Inactive UC AUPRC: 0.68	Diagnosis of UC	Validation using blood gene expression profiles is required. Restricted data and lack of diverse training/validation sets.
Li, 2020 [37]	-	RF, artificial neural network (ANN)	Gene Expression Profiles	UC	Best ANN model AUC: 0.9506	Diagnosis of UC	The validation set GSE92415 is relatively small.
Duttagupta, 2012 [38]	Recursive SVM	Recursive SVM	MicroRNAs	UC	SVM classifier accuracy: 0.92	Diagnosis of UC	RFE-SVM is computationally intensive. RFE was susceptible to overfitting in high-dimensional data.
Crohn’s Disease (CD)-Related Studies							
Raimondi, 2020 [39]	-	Neural network	Whole exomes	CD	AUC: 0.74–0.83 AUPRC: 0.81–0.93	Diagnosis of CD	Small sample size limitation.
Romagnoni, 2019 [40]	ResDN3 with Permutation feature importance (PFI), Lasso with weight as feature importance score, and LightGBM (LGBM) with gain	LR, gradient-boosted trees, neural network, and ensemble method	Genetics, Immunochip	CD	SNP-based model AUC: 0.80	Risk of CD	Limited to a finite set of algorithms.
Wang, 2019 [41]	-	Analysis of Variation for Association with Disease (AVADx) and two Genome-wide association studies (GWAS)-based CD evaluation methods	Whole Exome or Genome Sequencing Data	CD	AVADx model: - Identified known, new CD genes - 16% CD detection, 99% precision at strict cutoff - 58% CD detection, 82% precision at default cutoff	Diagnosis of CD	Low overlap between test and training data.
Bottigliengo, 2019 [42]	Bayesian machine learning techniques (BMLTs)	Generalized additive model (GAM), LR, linear discriminant analysis (LDA), quadratic discriminant analysis (QDA), projection pursuit regression (PPR), ANN, and BART.	Genetics	CD	PPR model with genetics AUC: 0.94	Diagnosis of CD	Missing data degraded performance. Simplistic Bayesian approach needs improvement.
Daneshjou, 2017 [43]	-	Naïve bayes, neural networks, random forests	Exome Sequencing	CD	Top methods AUC: 0.87	Diagnosis of CD	Small sample: 50 training, 53 testing.
Pal, 2017 [44]	-	Naïve Bayes	Genotypes from Exome Sequencing Data	CD	SNP model AUC for CD risk: 0.72	Risk of CD	No validation cohort
Cui and Zhang, 2013 [45]	Recursive SVM	Recursive SVM	16S rRNA gene analysis	CD/UC	LOOCV accuracy 0.88 with RSVM model.	Diagnosis of CD	More features increase complexity and training time. Only one Recursive FS method was used for biomarker selection.

The symbol “-” means data is unavailable.

**Table 2:** (**a**) List the top 10 upregulated genes compared to IBD and Healthy controls and (**b**) the top 10 downregulated genes between individuals with ITB and healthy controls.

(a)								
Gene	log2 Fold Change	*p*-Value	*q*-Value Calc	Mean Fold Change (gene_IBD)	Mean Fold Change (Control)	Fold Change Ratio	Fold Change Threshold	Category
*DENND2B*	0.114968	2.56 × 10^−23^	2.84 × 10^−19^	8.080239	7.461316	1.082951	1.06712	Upregulated
*LCN2*	0.495031	9.78 × 10^−23^	4.90 × 10^−19^	11.17161	7.926777	1.409351	1.06712	Upregulated
*IFITM3*	0.216846	2.20 × 10^−22^	9.14 × 10^−19^	8.908089	7.664914	1.16219	1.06712	Upregulated
*SLC6A14*	0.924287	9.60 × 10^−21^	2.46 × 10^−17^	9.582492	5.049408	1.897746	1.06712	Upregulated
*BACE2*	0.211402	1.49 × 10^−20^	3.31 × 10^−17^	10.00574	8.641933	1.157813	1.06712	Upregulated
*S100A11*	0.170625	4.61 × 10^−20^	9.01 × 10^−17^	10.38891	9.230108	1.125546	1.06712	Upregulated
*PLS3*	0.232287	2.92 × 10^−19^	4.86 × 10^−16^	7.948954	6.766822	1.174695	1.06712	Upregulated
*PARP8*	0.191193	1.81 × 10^−18^	2.51 × 10^−15^	8.399721	7.357159	1.141707	1.06712	Upregulated
*NFKBIZ*	0.210711	4.67 × 10^−18^	5.82 × 10^−15^	9.307241	8.042494	1.157258	1.06712	Upregulated
*DUOX2*	0.654203	4.72 × 10^−18^	5.82 × 10^−15^	11.23865	7.141333	1.573747	1.06712	Upregulated
**(b)**								
**Gene**	**log2 Fold Change**	***p*** **-Value**	***q*** **-Value Calc**	**Mean Fold Change (gene_IBD)**	**Mean Fold Change (Control)**	**Fold Change Ratio**	**Fold Change Threshold**	**Category**
*CNTFR*	−0.34736	1.91 × 10^−27^	6.35 × 10^−23^	7.287459	9.271327	0.786021	1.06712	Downregulated
*RNY1P5*	−0.53567	2.83 × 10^−24^	4.71 × 10^−20^	4.83799	7.013241	0.689837	1.06712	Downregulated
*STBD1*	−0.25467	3.97 × 10^−23^	3.30 × 10^−19^	6.584684	7.855963	0.838177	1.06712	Downregulated
*HINFP*	−0.11007	9.25 × 10^−23^	4.90 × 10^−19^	7.734588	8.34778	0.926544	1.06712	Downregulated
*PAQR5*	−0.34279	1.03 × 10^−22^	4.90 × 10^−19^	7.309534	9.270027	0.788513	1.06712	Downregulated
*CNTN4*	−0.28721	2.49 × 10^−22^	9.18 × 10^−19^	5.392263	6.580045	0.819487	1.06712	Downregulated
*RNF135*	−0.09782	7.81 × 10^−22^	2.60 × 10^−18^	8.14145	8.712626	0.934443	1.06712	Downregulated
*FRMD1*	−0.24535	1.16 × 10^−21^	3.51 × 10^−18^	6.332201	7.506043	0.843614	1.06712	Downregulated
*SFXN1*	−0.12522	4.85 × 10^−21^	1.34 × 10^−17^	8.743877	9.536722	0.916864	1.06712	Downregulated
*SLC38A4*	−0.39528	1.35 × 10^−20^	3.21 × 10^−17^	4.856832	6.387714	0.76034	1.06712	Downregulated

**Table 3:** List upregulated and downregulated features selected using six fs algorithms.

FS Methods	Type of FS Method	Selected Feature (s)	
		Upregulated	Downregulated
Mutual Information Score	Filter	7960464 (*VWF*)	8101086 (*NAAA*)
RFECV	Wrapper	7946401 (*DENND2B*)	8101086 (*NAAA*)
Elastic Net	Embedded	8044021 (*IL1RL1*)	7934945 (*PANK1*)
Gradient Boosting Classifier	Embedded	7973336 (*MMP14*)	8101086 (*NAAA*)

**Table 4:** The master subset of gene biomarkers for classifying IBD from non-IBD samples.

Selected Gene Biomarkers
7960464 (*VWF*), 7946401 (*DENND2B*), 8044021 (*IL1RL1*), 7973336 (*MMP14*), 7934945 (*PANK1*), 8101086 (*NAAA*)

**Table 5:** Presents the results of an unpaired t-test to assess potential gene biomarkers’ ability to classify two classes.

Potential Gene Biomarkers	IBD Mean	IBD Std	Normal Mean	Normal Std	*t*-Statistic	*p*-Value
*VWF*	7.96	0.882	6.528	0.4260	7.522	2.02 × 10^−12^
*IL1RL1*	5.59	0.541	5.095	0.276	4.223	3.72 × 10^−5^
*DENND2B*	8.08	0.241	7.461	0.232	11.397	2.56 × 10^−23^
*MMP14*	8.83	0.525	8.048	0.250	6.961	5.21 × 10^−11^
*NAAA*	9.09	0.686	10.384	0.241	−8.718	1.32 × 10^−15^
*PANK1*	7.95	0.485	8.563	0.253	−5.758	3.32 × 10^−8^

**Table 6:** Compares the performance of seven classification models using the five most informative features to baseline models using all features of the test/validation E-GEOD-36807 dataset.

ML Algorithms	Accuracy (SMOTE)	Accuracy (No SMOTE)	F1 Score (SMOTE)	F1 Score (No SMOTE)	AUC (SMOTE)	AUC (No SMOTE)	Baseline Accuracy (No SMOTE)	Baseline F1 Score (No SMOTE)	Baseline AUC (No SMOTE)
GNB	0.9680 ± 0.0061	0.94331 ± 0.01912	0.9674 ± 0.0055	0.96732 ± 0.01126	0.9680 ± 0.0061	0.9238 ± 0.0585	0.8713 ± 0.0666	0.5930 ± 0.1811	0.83899 ± 0.1299
RF	0.9767 ± 0.0148	0.9532 ± 0.0454	0.97628 ± 0.0150	0.9740 ± 0.0250	0.9767 ± 0.0148	0.86840 ± 0.1460	0.9277 ± 0.0305	0.5024 ± 0.3075	0.7021 ± 0.1356
LR	0.9667 ± 0.0150	0.9584 ± 0.0357	0.9663 ± 0.0152	0.96706 ± 0.01934	0.9768 ± 0.0149	0.87134 ± 0.1405	0.9432 ± 0.0302	0.6458 ± 0.2335	0.7771 ± 0.1471
MLP	0.9360 ± 0.0235	0.9380 ± 0.0354	0.9324 ± 0.0264	0.96626 ± 0.0185	0.9362 ± 0.0230	0.7693 ± 0.1863	0.578947 ± 0.3787	0.082663 ± 0.101	0.5 ± 0.0
SVC	0.9738 ± 0.01086	0.9584 ± 0.0357	0.9731 ± 0.0112	0.97706 ± 0.0193	0.9739 ± 0.0106	0.87134 ± 0.1405	0.8866 ± 0.0121	0.0 ± 0.0	0.5 ± 0.0
DT	0.9622 ± 0.0217	0.9275 ± 0.0351	0.9615 ± 0.0220	0.9599 ± 0.0191	0.9623 ± 0.0218	0.7705 ± 0.1265	0.8659 ± 0.0679	0.450 ± 0.2082	0.6846 ± 0.1205
XGB	0.92766 ± 0.0305	0.9277 ± 0.0305	0.9596 ± 0.0168	0.9596 ± 0.0168	0.8026 ± 0.1122	0.8026 ± 0.1122	0.9381 ± 0.0208	0.6554 ± 0.1648	0.7813 ± 0.0968

**Table 7:** The gene ontology and pathway enrichment analysis outcomes performed on the four upregulated DEGs.

Sl. No.	Term/Pathway	*p*-Value	Genes
Gene Ontology			
1	GO:0050867; positive regulation of cell activation	0.0013070	*IL1RL1* and *MMP14*
2	GO:0002694; regulation of leukocyte activation	0.0031340	*IL1RL1* and *MMP14*
3	GO:0043062; extracellular structure organization	0.0022000	*VWF* and *MMP14*
4	GO:0031589; cell-substrate adhesion	0.0015271	*VWF* and *MMP14*
5	GO:0032634; interleukin-5 production	0.0045196	*IL1RL1*
KEGG Pathway			
1	hsa04610; Complement and coagulation cascades	0.032090	*VWF*
2	hsa04512; ECM-receptor interaction	0.033294	*VWF*
3	hsa04912; GnRH signaling pathway	0.037303	*MMP14*
4	hsa04928; secretion and action, Parathyroid hormone synthesis	0.043109	*MMP14*
5	hsa04668; TNF signaling pathway	0.044093	*MMP14*
6	hsa04611; Platelet activation	0.049661	*VWF*
REACTOME Pathway			
1	R-HSA-430116; GP1b-IX-V activation signaling	0.0034394	*VWF*
2	R-HSA-140837; Intrinsic Pathway of Fibrin Clot Formation	0.0062995	*VWF*
3	R-HSA-372708; p130Cas linkage to MAPK signaling for integrins	0.0042980	*VWF*, *DENND2B*
4	R-HSA-9006921; Integrin signaling	0.0077275	*VWF*
5	R-HSA-75892; Platelet Adhesion to Exposed Collagen	0.0042980	*VWF*
6	R-HSA-354194; GRB2: SOS provides linkage to MAPK signaling for Integrins	0.0042980	*VWF*, *DENND2B*
7	R-HSA-1474244; Extracellular matrix organization	0.0023830	*MMP14* and *VWF*
8	R-HSA-1592389; Activation of Matrix Metalloproteinases	0.0094393	*MMP14*
9	R-HSA-6802948; Signaling by high-kinase activity BRAF mutants	0.010294	*VWF*

**Table 8:** Listing the outcomes of the gene ontology and pathway enrichment analysis performed on the two downregulated DEGs.

Sl. No.	Term/Pathway	*p*-Value	Genes
Gene Ontology			
1	GO:0033865; nucleoside bisphosphate metabolic process	0.018431	*PANK1*
2	GO:0072522; purine-containing compound biosynthetic process	0.036017	*PANK1*
3	GO:0046390; ribose phosphate biosynthetic process	0.034400	*PANK1*
4	GO:0006836; neurotransmitter transport	0.036286	*NAAA*
5	GO:0051188; cofactor biosynthetic process	0.037767	*PANK1*
6	GO:1901293; nucleoside phosphate biosynthetic process	0.046225	*PANK1*
7	GO:0006732; coenzyme metabolic process	0.047564	*PANK1*
8	GO:0001505; regulation of neurotransmitter levels	0.045287	*NAAA*
KEGG Pathway			
1	hsa00770; Pantothenate and CoA biosynthesis	0.0026002	*PANK1*
REACTOME Pathway			
1	R-HSA-199220; Vitamin B5 (pantothenate) metabolism	0.0032492	*PANK1*
2	R-HSA-196783; Coenzyme A biosynthesis	0.0015297	*PANK1*
3	R-HSA-112315; Transmission across Chemical Synapses	0.042764	*NAAA*
4	R-HSA-112310; Neurotransmitter release cycle	0.0097318	*NAAA*
5	R-HSA-196854; Metabolism of vitamins and cofactors	0.035826	*PANK1*
6	R-HSA-196849; Metabolism of water-soluble vitamins and cofactors	0.023390	*PANK1*

## Data Availability

Datasets are publicly available at: https://www.ncbi.nlm.nih.gov/geo/, accessed on 1 December 2023.

## Supplementary Materials

The following supporting information can be downloaded at: https://www.mdpi.com/article/10.3390/diagnostics14111182/s1, Table S1: List the total upregulated genes in comparison between IBD and healthy controls in GSE75214 data; Table S2: The total downregulated genes in comparison between individuals with IBD and healthy controls in GSE75214 data.
