# Supplementary material for: Advances in Inflammatory Bowel Disease Diagnostics: Machine Learning and Genomic Profiling Reveal Key Biomarkers for Early Detection
Source: Diagnostics (Basel). 2024 Jun 4;14(11):1182. doi: 10.3390/diagnostics14111182 (PMC11172026; doi:10.3390/diagnostics14111182)
Supplement: Supplementary file 1 [file diagnostics-14-01182-s001.zip › diagnostics-3006751-supplementary.pdf]

Advances in Inflammatory Bowel Disease Diagnostics: Machine Learning and Genomic Profiling Reveal Key Biomarkers for Early Detection

SUPPLEMENTARY TABLE S1 AND S2

Table S1: LIST THE TOTAL UPREGULATED GENES IN COMPARISON BETWEEN IBD AND HEALTHY CONTROLS IN GSE75214 DATA

Gene	log2_fold_change	p-value	q-value calc	Mean Fold Change (gene_IBD)	Mean Fold Change (control)	Fold Change Ratio	Fold Change Threshold	category
**7946401**	0.114968	2.56E-23	2.84E-19	8.080239	7.461316	1.082951	1.06712	Upregulated
**8158167**	0.495031	9.78E-23	4.90E-19	11.17161	7.926777	1.409351	1.06712	Upregulated
**7945371**	0.216846	2.20E-22	9.14E-19	8.908089	7.664914	1.16219	1.06712	Upregulated
**8169504**	0.924287	9.60E-21	2.46E-17	9.582492	5.049408	1.897746	1.06712	Upregulated
**8068671**	0.211402	1.49E-20	3.31E-17	10.00574	8.641933	1.157813	1.06712	Upregulated
**7920128**	0.170625	4.61E-20	9.01E-17	10.38891	9.230108	1.125546	1.06712	Upregulated
**8169473**	0.232287	2.92E-19	4.86E-16	7.948954	6.766822	1.174695	1.06712	Upregulated
**8105191**	0.191193	1.81E-18	2.51E-15	8.399721	7.357159	1.141707	1.06712	Upregulated
**8081386**	0.210711	4.67E-18	5.82E-15	9.307241	8.042494	1.157258	1.06712	Upregulated
**7988350**	0.654203	4.72E-18	5.82E-15	11.23865	7.141333	1.573747	1.06712	Upregulated

Table S2: THE TOTAL DOWNREGULATED GENES IN COMPARISON BETWEEN INDIVIDUALS WITH ITB AND HEALTHY CONTROLS IN GSE75214 DATA

Gene	log2_fold_change	p-value	q-value calc	Mean Fold Change (gene_IBD)	Mean Fold Change (control)	s	Fold Change Threshold	category
**8160823**	-0.34736	1.91E-27	6.35E-23	7.287459	9.271327	0.786021	1.06712	Downregulated
**7972016**	-0.53567	2.83E-24	4.71E-20	4.83799	7.013241	0.689837	1.06712	Downregulated
**8095826**	-0.25467	3.97E-23	3.30E-19	6.584684	7.855963	0.838177	1.06712	Downregulated
**7944435**	-0.11007	9.25E-23	4.90E-19	7.734588	8.34778	0.926544	1.06712	Downregulated
**7984524**	-0.34279	1.03E-22	4.90E-19	7.309534	9.270027	0.788513	1.06712	Downregulated
**8077323**	-0.28721	2.49E-22	9.18E-19	5.392263	6.580045	0.819487	1.06712	Downregulated
**8006229**	-0.09782	7.81E-22	2.60E-18	8.14145	8.712626	0.934443	1.06712	Downregulated
**8130837**	-0.24535	1.16E-21	3.51E-18	6.332201	7.506043	0.843614	1.06712	Downregulated
**8110090**	-0.12522	4.85E-21	1.34E-17	8.743877	9.536722	0.916864	1.06712	Downregulated
**7962559**	-0.39528	1.35E-20	3.21E-17	4.856832	6.387714	0.76034	1.06712	Downregulated
**8002556**	-0.22594	2.57E-20	5.33E-17	5.380388	6.292584	0.855036	1.06712	Downregulated
**7983661**	-0.37767	7.42E-20	1.37E-16	4.24755	5.518602	0.769679	1.06712	Downregulated
**8096905**	-0.12641	8.30E-19	1.25E-15	7.830639	8.547743	0.916106	1.06712	Downregulated
**8162194**	-0.18107	1.18E-18	1.70E-15	6.325209	7.171061	0.882046	1.06712	Downregulated

**8151512**	-0.15085	3.24E-18	4.31E-15	8.515987	9.454673	0.900717	1.06712	Downregulated
**8180361**	-0.15462	1.00E-17	1.19E-14	7.880437	8.771962	0.898366	1.06712	Downregulated
**8105456**	-0.21003	2.51E-17	2.53E-14	5.864562	6.783603	0.86452	1.06712	Downregulated
**8095819**	-0.22196	3.45E-17	3.38E-14	4.91377	5.731032	0.857397	1.06712	Downregulated
**8025964**	-0.14004	1.15E-16	9.33E-14	5.693933	6.274362	0.907492	1.06712	Downregulated
**8057821**	-0.16725	1.43E-16	1.13E-13	5.004257	5.619363	0.890538	1.06712	Downregulated
**7995829**	-0.27229	1.79E-16	1.33E-13	8.724794	10.53717	0.828001	1.06712	Downregulated
**7900531**	-0.27715	2.66E-16	1.88E-13	9.292105	11.26015	0.82522	1.06712	Downregulated
**8004152**	-0.18247	4.52E-16	3.01E-13	6.936052	7.871214	0.881192	1.06712	Downregulated
**8000700**	-0.20144	4.93E-16	3.15E-13	4.200843	4.830305	0.869685	1.06712	Downregulated
**8003599**	-0.20144	4.93E-16	3.15E-13	4.200843	4.830305	0.869685	1.06712	Downregulated
**7995787**	-0.34853	5.29E-16	3.26E-13	8.459507	10.77118	0.785383	1.06712	Downregulated
**7903162**	-0.23818	5.30E-16	3.26E-13	8.1623	9.627454	0.847815	1.06712	Downregulated
**7995803**	-0.20106	7.30E-16	4.34E-13	7.213153	8.291806	0.869913	1.06712	Downregulated
**7975932**	-0.2827	1.06E-15	6.02E-13	5.823101	7.083623	0.822051	1.06712	Downregulated
**7928119**	-0.09599	1.08E-15	6.02E-13	10.76359	11.50414	0.935627	1.06712	Downregulated
**8049317**	-0.09763	1.09E-15	6.02E-13	9.035286	9.667854	0.93457	1.06712	Downregulated

**8101086**	-0.19115	1.32E-15	7.09E-13	9.09557	10.38416	0.875908	1.06712	Downregulated
**7942061**	-0.23531	1.39E-15	7.34E-13	6.719118	7.9095	0.8495	1.06712	Downregulated
**8111101**	-0.12957	1.53E-15	7.93E-13	8.574744	9.380524	0.914101	1.06712	Downregulated
**8006237**	-0.13907	1.57E-15	8.01E-13	7.300056	8.038773	0.908106	1.06712	Downregulated
**8121225**	-0.1306	1.93E-15	9.72E-13	5.00878	5.483367	0.91345	1.06712	Downregulated
**8130422**	-0.23538	2.99E-15	1.40E-12	7.140957	8.406459	0.849461	1.06712	Downregulated
**7939184**	-0.13176	6.13E-15	2.75E-12	7.275276	7.970995	0.912719	1.06712	Downregulated
**7903334**	-0.24132	6.51E-15	2.89E-12	7.875583	9.309503	0.845972	1.06712	Downregulated
**7983928**	-0.17371	6.83E-15	2.99E-12	5.058467	5.705742	0.886557	1.06712	Downregulated
**8054997**	-0.09525	8.08E-15	3.44E-12	8.596177	9.182864	0.936111	1.06712	Downregulated
**8157253**	-0.13344	1.02E-14	4.24E-12	8.090157	8.874157	0.911654	1.06712	Downregulated
**8002878**	-0.10005	1.53E-14	6.04E-12	9.940522	10.65438	0.932999	1.06712	Downregulated
**7995813**	-0.13372	1.70E-14	6.38E-12	8.853466	9.71333	0.911476	1.06712	Downregulated
**7901634**	-0.1464	1.71E-14	6.38E-12	6.368009	7.048138	0.903502	1.06712	Downregulated
**7995806**	-0.16762	1.86E-14	6.80E-12	9.004206	10.11357	0.890309	1.06712	Downregulated
**7911138**	-0.09916	1.86E-14	6.80E-12	7.278133	7.795994	0.933573	1.06712	Downregulated
**7943729**	-0.15479	2.21E-14	7.91E-12	5.714764	6.362031	0.898261	1.06712	Downregulated

**7921492**	-0.17209	2.43E-14	8.58E-12	7.732513	8.712143	0.887556	1.06712	Downregulated
**8105949**	-0.13667	2.57E-14	8.82E-12	6.97059	7.663198	0.909619	1.06712	Downregulated
**8105997**	-0.13667	2.57E-14	8.82E-12	6.97059	7.663198	0.909619	1.06712	Downregulated
**8177658**	-0.13667	2.57E-14	8.82E-12	6.97059	7.663198	0.909619	1.06712	Downregulated
**8106689**	-0.12095	5.16E-14	1.65E-11	5.681532	6.178381	0.919583	1.06712	Downregulated
**8059538**	-0.27959	6.01E-14	1.87E-11	6.381407	7.746051	0.823827	1.06712	Downregulated
**8062119**	-0.16728	6.82E-14	2.08E-11	8.131342	9.131024	0.890518	1.06712	Downregulated
**8090664**	-0.13273	7.13E-14	2.16E-11	6.479734	7.104142	0.912107	1.06712	Downregulated
**8127370**	-0.15	7.55E-14	2.26E-11	5.25613	5.832062	0.901247	1.06712	Downregulated
**7995820**	-0.126	7.77E-14	2.31E-11	7.689528	8.391291	0.91637	1.06712	Downregulated
**8083447**	-0.13955	9.38E-14	2.70E-11	7.465787	8.224024	0.907802	1.06712	Downregulated
**7905929**	-0.19187	9.41E-14	2.70E-11	7.559778	8.635128	0.875468	1.06712	Downregulated
**8011354**	-0.22352	9.88E-14	2.81E-11	6.437125	7.515839	0.856475	1.06712	Downregulated
**8103524**	-0.10271	1.08E-13	2.97E-11	7.079037	7.601368	0.931285	1.06712	Downregulated
**8138741**	-0.21944	1.24E-13	3.36E-11	7.493723	8.724796	0.8589	1.06712	Downregulated
**8091446**	-0.2177	1.26E-13	3.38E-11	7.808548	9.080394	0.859935	1.06712	Downregulated
**7925413**	-0.11276	1.27E-13	3.39E-11	8.021517	8.673652	0.924814	1.06712	Downregulated

**8178676**	-0.10801	1.44E-13	3.72E-11	8.983193	9.681526	0.92787	1.06712	Downregulated
**8125139**	-0.10207	1.78E-13	4.43E-11	8.61125	9.242547	0.931697	1.06712	Downregulated
**8179851**	-0.10207	1.78E-13	4.43E-11	8.61125	9.242547	0.931697	1.06712	Downregulated
**8078187**	-0.10131	1.79E-13	4.43E-11	7.456141	7.998538	0.932188	1.06712	Downregulated
**8080306**	-0.14256	1.81E-13	4.43E-11	6.628104	7.316501	0.905912	1.06712	Downregulated
**8112182**	-0.16206	1.99E-13	4.70E-11	9.049627	10.12548	0.893748	1.06712	Downregulated
**8008172**	-0.32437	2.15E-13	4.93E-11	7.105783	8.897252	0.798649	1.06712	Downregulated
**8107920**	-0.23958	2.38E-13	5.38E-11	8.524237	10.06411	0.846994	1.06712	Downregulated
**8110932**	-0.16195	2.46E-13	5.48E-11	8.680119	9.711284	0.893818	1.06712	Downregulated
**8008321**	-0.28162	2.69E-13	5.88E-11	7.833553	9.522136	0.822668	1.06712	Downregulated
**8133961**	-0.2584	2.94E-13	6.40E-11	6.356068	7.602829	0.836013	1.06712	Downregulated
**8023598**	-0.20956	3.30E-13	6.95E-11	7.816752	9.038794	0.8648	1.06712	Downregulated
**7969488**	-0.09775	3.80E-13	7.85E-11	7.930884	8.486884	0.934487	1.06712	Downregulated
**8052872**	-0.11933	4.61E-13	9.08E-11	6.996314	7.599624	0.920613	1.06712	Downregulated
**7978558**	-0.09743	4.68E-13	9.16E-11	7.902947	8.455073	0.934699	1.06712	Downregulated
**7995838**	-0.17431	5.94E-13	1.12E-10	9.211939	10.39501	0.886189	1.06712	Downregulated
**7928770**	-0.36532	6.40E-13	1.18E-10	7.193722	9.266688	0.776299	1.06712	Downregulated

**8149551**	-0.13855	8.40E-13	1.49E-10	7.452613	8.203833	0.908431	1.06712	Downregulated
**7943349**	-0.13121	8.75E-13	1.54E-10	7.840486	8.587014	0.913063	1.06712	Downregulated
**8012908**	-0.11315	9.08E-13	1.58E-10	5.016793	5.426103	0.924566	1.06712	Downregulated
**7975926**	-0.12986	9.72E-13	1.67E-10	7.751692	8.481806	0.91392	1.06712	Downregulated
**8171725**	-0.15051	1.16E-12	1.90E-10	5.362136	5.951769	0.900931	1.06712	Downregulated
**7995834**	-0.13664	1.19E-12	1.92E-10	7.053397	7.75409	0.909636	1.06712	Downregulated
**8063071**	-0.10587	1.35E-12	2.12E-10	6.32763	6.809413	0.929247	1.06712	Downregulated
**8132439**	-0.223	1.37E-12	2.14E-10	6.054157	7.066182	0.856779	1.06712	Downregulated
**8043310**	-0.13704	1.43E-12	2.22E-10	8.625668	9.485172	0.909384	1.06712	Downregulated
**7958439**	-0.12172	1.45E-12	2.25E-10	8.570516	9.325017	0.919089	1.06712	Downregulated
**8144947**	-0.1277	1.46E-12	2.25E-10	5.229676	5.713674	0.915291	1.06712	Downregulated
**8066609**	-0.11784	1.62E-12	2.46E-10	6.495932	7.048776	0.921569	1.06712	Downregulated
**7956018**	-0.12431	1.67E-12	2.51E-10	7.292554	7.948757	0.917446	1.06712	Downregulated
**8109161**	-0.12158	1.88E-12	2.78E-10	6.765416	7.360293	0.919178	1.06712	Downregulated
**8002571**	-0.25775	1.92E-12	2.81E-10	8.727057	10.43418	0.836392	1.06712	Downregulated
**7924092**	-0.10247	2.20E-12	3.17E-10	8.35672	8.971876	0.931435	1.06712	Downregulated
**7995976**	-0.12254	2.53E-12	3.56E-10	9.451095	10.28891	0.918571	1.06712	Downregulated

**7938519**	-0.15496	2.56E-12	3.60E-10	6.647107	7.400853	0.898154	1.06712	Downregulated
**7905789**	-0.0959	2.91E-12	4.05E-10	8.447246	9.027852	0.935687	1.06712	Downregulated
**8076355**	-0.13076	3.07E-12	4.25E-10	7.431809	8.136891	0.913347	1.06712	Downregulated
**8008263**	-0.20724	3.09E-12	4.26E-10	8.408816	9.707809	0.866191	1.06712	Downregulated
**7935230**	-0.10654	3.23E-12	4.40E-10	9.108351	9.806465	0.928811	1.06712	Downregulated
**8071136**	-0.10243	3.35E-12	4.51E-10	9.118934	9.789899	0.931464	1.06712	Downregulated
**7932094**	-0.16405	3.40E-12	4.54E-10	7.770114	8.705876	0.892514	1.06712	Downregulated
**8028963**	-0.44263	3.43E-12	4.56E-10	7.47752	10.16252	0.735794	1.06712	Downregulated
**8082869**	-0.19306	3.79E-12	4.97E-10	6.484762	7.413283	0.874749	1.06712	Downregulated
**8001531**	-0.17226	3.92E-12	5.11E-10	11.43008	12.87964	0.887453	1.06712	Downregulated
**8111430**	-0.16005	3.98E-12	5.17E-10	7.945307	8.877485	0.894995	1.06712	Downregulated
**7995825**	-0.18566	4.23E-12	5.39E-10	11.06343	12.58289	0.879244	1.06712	Downregulated
**7933228**	-0.11543	4.78E-12	5.94E-10	8.943674	9.688637	0.92311	1.06712	Downregulated
**7971369**	-0.10126	4.93E-12	6.05E-10	5.006712	5.37076	0.932217	1.06712	Downregulated
**8009746**	-0.10539	5.11E-12	6.25E-10	7.803962	8.395401	0.929552	1.06712	Downregulated
**8130811**	-0.15904	5.37E-12	6.42E-10	5.740811	6.409886	0.895618	1.06712	Downregulated
**8130982**	-0.15904	5.37E-12	6.42E-10	5.740811	6.409886	0.895618	1.06712	Downregulated

**8093518**	-0.14313	5.57E-12	6.60E-10	7.838855	8.656424	0.905553	1.06712	Downregulated
**7991323**	-0.11288	5.62E-12	6.63E-10	7.132915	7.71343	0.92474	1.06712	Downregulated
**7983890**	-0.14445	6.50E-12	7.53E-10	7.220766	7.981175	0.904725	1.06712	Downregulated
**8014349**	-0.10175	6.53E-12	7.53E-10	8.442782	9.059737	0.931901	1.06712	Downregulated
**8115041**	-0.21611	6.68E-12	7.66E-10	5.17528	6.01161	0.860881	1.06712	Downregulated
**8009705**	-0.31829	7.11E-12	8.01E-10	7.96626	9.932762	0.802019	1.06712	Downregulated
**7995797**	-0.11964	7.14E-12	8.02E-10	9.598532	10.42843	0.92042	1.06712	Downregulated
**8124262**	-0.15395	7.18E-12	8.04E-10	9.625356	10.70931	0.898784	1.06712	Downregulated
**8021058**	-0.18002	7.28E-12	8.09E-10	5.831523	6.606514	0.882693	1.06712	Downregulated
**8006085**	-0.13443	7.61E-12	8.38E-10	4.665288	5.120885	0.911032	1.06712	Downregulated
**8096070**	-0.32365	7.91E-12	8.65E-10	7.385192	9.242487	0.799048	1.06712	Downregulated
**8016718**	-0.20879	8.19E-12	8.92E-10	7.190071	8.309678	0.865265	1.06712	Downregulated
**8165552**	-0.1069	8.59E-12	9.27E-10	7.747256	8.343085	0.928584	1.06712	Downregulated
**7902367**	-0.13198	9.60E-12	1.02E-09	9.519736	10.43172	0.912576	1.06712	Downregulated
**8000890**	-0.09451	9.75E-12	1.03E-09	9.277172	9.905283	0.936588	1.06712	Downregulated
**8010021**	-0.11396	1.01E-11	1.06E-09	6.707111	7.25839	0.924049	1.06712	Downregulated
**8004081**	-0.11622	1.35E-11	1.35E-09	7.134516	7.733058	0.9226	1.06712	Downregulated

**8047487**	-0.14398	1.38E-11	1.38E-09	6.868555	7.589379	0.905022	1.06712	Downregulated
**8112107**	-0.1052	1.39E-11	1.38E-09	8.867441	9.538226	0.929674	1.06712	Downregulated
**8014871**	-0.11219	1.40E-11	1.38E-09	8.071769	8.724506	0.925183	1.06712	Downregulated
**8138718**	-0.17581	1.74E-11	1.66E-09	6.505848	7.349017	0.885268	1.06712	Downregulated
**7997491**	-0.21926	1.80E-11	1.69E-09	8.6983	10.126	0.859007	1.06712	Downregulated
**7957221**	-0.33076	1.80E-11	1.69E-09	5.744676	7.224961	0.795115	1.06712	Downregulated
**8107673**	-0.13637	1.83E-11	1.70E-09	8.612329	9.46614	0.909804	1.06712	Downregulated
**7975203**	-0.13755	2.03E-11	1.87E-09	8.040582	8.844927	0.909061	1.06712	Downregulated
**8162570**	-0.21996	2.06E-11	1.90E-09	5.587497	6.507756	0.858591	1.06712	Downregulated
**8149250**	-0.11422	2.14E-11	1.95E-09	7.970101	8.626769	0.92388	1.06712	Downregulated
**8064388**	-0.16644	2.24E-11	2.03E-09	8.212056	9.216277	0.891038	1.06712	Downregulated
**8160452**	-0.18144	2.38E-11	2.13E-09	8.720019	9.88866	0.88182	1.06712	Downregulated
**8067029**	-0.13547	2.48E-11	2.21E-09	6.704621	7.364704	0.910372	1.06712	Downregulated
**8098084**	-0.18233	2.50E-11	2.22E-09	8.159772	9.258994	0.881281	1.06712	Downregulated
**7973135**	-0.10816	2.70E-11	2.37E-09	4.962145	5.348447	0.927773	1.06712	Downregulated
**8120315**	-0.25729	3.15E-11	2.69E-09	6.785306	8.110024	0.836657	1.06712	Downregulated
**7918913**	-0.14528	3.24E-11	2.76E-09	7.788233	8.613378	0.904202	1.06712	Downregulated

**8138735**	-0.15424	3.31E-11	2.80E-09	7.141221	7.947027	0.898603	1.06712	Downregulated
**8173600**	-0.14287	3.41E-11	2.85E-09	4.371614	4.8267	0.905715	1.06712	Downregulated
**8059071**	-0.12427	3.70E-11	3.05E-09	7.417339	8.084557	0.91747	1.06712	Downregulated
**7974781**	-0.09787	3.73E-11	3.07E-09	8.027241	8.590692	0.934411	1.06712	Downregulated
**8024532**	-0.12572	4.08E-11	3.33E-09	7.936168	8.658799	0.916544	1.06712	Downregulated
**8118535**	-0.12037	4.25E-11	3.44E-09	7.974622	8.668533	0.919951	1.06712	Downregulated
**8179472**	-0.12037	4.25E-11	3.44E-09	7.974622	8.668533	0.919951	1.06712	Downregulated
**8000899**	-0.09864	4.66E-11	3.71E-09	7.600971	8.138826	0.933915	1.06712	Downregulated
**7943369**	-0.11881	5.30E-11	4.16E-09	7.571195	8.221073	0.92095	1.06712	Downregulated
**8138721**	-0.11204	5.33E-11	4.17E-09	6.844909	7.397658	0.925281	1.06712	Downregulated
**7923608**	-0.10366	5.49E-11	4.24E-09	5.385295	5.78648	0.930669	1.06712	Downregulated
**8047062**	-0.15553	5.80E-11	4.46E-09	7.860169	8.754904	0.897802	1.06712	Downregulated
**8088602**	-0.11271	5.89E-11	4.51E-09	8.070337	8.726112	0.924849	1.06712	Downregulated
**8021768**	-0.10411	6.21E-11	4.74E-09	7.617906	8.187974	0.930377	1.06712	Downregulated
**8101624**	-0.11179	6.40E-11	4.87E-09	7.001336	7.565412	0.92544	1.06712	Downregulated
**8015115**	-0.20056	6.47E-11	4.90E-09	6.101275	7.011262	0.870211	1.06712	Downregulated
**8028955**	-0.39363	6.48E-11	4.90E-09	6.503166	8.543155	0.761214	1.06712	Downregulated

**8161774**	-0.32594	6.50E-11	4.90E-09	7.50866	9.411952	0.797779	1.06712	Downregulated
**8122701**	-0.13509	6.90E-11	5.16E-09	6.041298	6.634305	0.910615	1.06712	Downregulated
**8150225**	-0.10016	6.92E-11	5.16E-09	8.670865	9.29424	0.932929	1.06712	Downregulated
**8062123**	-0.09582	7.02E-11	5.18E-09	6.205534	6.631673	0.935742	1.06712	Downregulated
**8100202**	-0.27604	7.39E-11	5.41E-09	5.110903	6.188602	0.825857	1.06712	Downregulated
**8078395**	-0.1933	7.78E-11	5.65E-09	5.594986	6.397167	0.874604	1.06712	Downregulated
**7932765**	-0.13025	8.11E-11	5.86E-09	7.65498	8.378242	0.913674	1.06712	Downregulated
**8022803**	-0.09395	8.13E-11	5.87E-09	8.434937	9.002497	0.936955	1.06712	Downregulated
**7901140**	-0.10706	8.30E-11	5.97E-09	9.217372	9.927398	0.928478	1.06712	Downregulated
**8121768**	-0.18626	8.33E-11	5.98E-09	7.231889	8.228536	0.878879	1.06712	Downregulated
**8178188**	-0.11238	8.35E-11	5.98E-09	9.10225	9.839621	0.925061	1.06712	Downregulated
**8164008**	-0.10223	8.84E-11	6.30E-09	6.405863	6.876254	0.931592	1.06712	Downregulated
**8042283**	-0.10605	8.88E-11	6.30E-09	9.429038	10.14827	0.929128	1.06712	Downregulated
**8122099**	-0.18166	9.42E-11	6.61E-09	7.775216	8.818528	0.881691	1.06712	Downregulated
**8150356**	-0.11814	9.64E-11	6.72E-09	7.795381	8.460623	0.921372	1.06712	Downregulated
**8023220**	-0.11191	1.07E-10	7.33E-09	7.275182	7.861967	0.925364	1.06712	Downregulated
**8157092**	-0.14908	1.12E-10	7.64E-09	6.819898	7.562316	0.901827	1.06712	Downregulated

**7904421**	-0.32489	1.15E-10	7.79E-09	6.220949	7.792155	0.798361	1.06712	Downregulated
**8113717**	-0.33904	1.16E-10	7.83E-09	4.978761	6.297688	0.79057	1.06712	Downregulated
**8041519**	-0.21966	1.21E-10	8.14E-09	6.407644	7.461453	0.858766	1.06712	Downregulated
**7935011**	-0.10082	1.25E-10	8.32E-09	7.001885	7.50871	0.932502	1.06712	Downregulated
**8053025**	-0.36091	1.33E-10	8.73E-09	6.245056	8.02011	0.778675	1.06712	Downregulated
**7997166**	-0.17261	1.35E-10	8.85E-09	4.789452	5.39818	0.887235	1.06712	Downregulated
**7969815**	-0.15293	1.40E-10	9.09E-09	7.48527	8.322328	0.89942	1.06712	Downregulated
**8072870**	-0.0995	1.40E-10	9.12E-09	8.5073	9.114739	0.933356	1.06712	Downregulated
**7980080**	-0.21177	1.45E-10	9.34E-09	8.856718	10.25706	0.863476	1.06712	Downregulated
**7910387**	-0.15231	1.45E-10	9.34E-09	9.226654	10.25401	0.89981	1.06712	Downregulated
**8058203**	-0.09835	1.53E-10	9.76E-09	5.358183	5.736187	0.934102	1.06712	Downregulated
**7966003**	-0.11858	1.54E-10	9.81E-09	9.274299	10.06876	0.921097	1.06712	Downregulated
**8083850**	-0.1925	1.56E-10	9.88E-09	6.165989	7.046137	0.875088	1.06712	Downregulated
**8046836**	-0.14711	1.76E-10	1.09E-08	4.353421	4.820743	0.90306	1.06712	Downregulated
**8073062**	-0.218	2.01E-10	1.24E-08	7.641843	8.888365	0.859758	1.06712	Downregulated
**7984245**	-0.24098	2.06E-10	1.26E-08	8.869567	10.48198	0.846173	1.06712	Downregulated
**7958262**	-0.10988	2.25E-10	1.36E-08	8.073519	8.712422	0.926667	1.06712	Downregulated

**7959267**	-0.11256	2.27E-10	1.37E-08	8.494364	9.183655	0.924944	1.06712	Downregulated
**7964555**	-0.11404	2.50E-10	1.49E-08	6.877801	7.443546	0.923995	1.06712	Downregulated
**7974047**	-0.13077	2.50E-10	1.49E-08	7.626633	8.35026	0.913341	1.06712	Downregulated
**8155554**	-0.21136	2.69E-10	1.59E-08	6.567109	7.603268	0.863722	1.06712	Downregulated
**8161488**	-0.20849	2.70E-10	1.60E-08	6.585719	7.609655	0.865443	1.06712	Downregulated
**7995793**	-0.12173	2.75E-10	1.62E-08	9.276654	10.09333	0.919087	1.06712	Downregulated
**8111151**	-0.10572	2.75E-10	1.62E-08	5.604349	6.030451	0.929342	1.06712	Downregulated
**8117207**	-0.12391	3.17E-10	1.84E-08	6.572977	7.162462	0.917698	1.06712	Downregulated
**8155442**	-0.21101	3.31E-10	1.90E-08	6.636846	7.682145	0.863931	1.06712	Downregulated
**8131919**	-0.11788	3.46E-10	1.97E-08	6.525966	7.081564	0.921543	1.06712	Downregulated
**8073826**	-0.10262	3.59E-10	2.03E-08	8.394025	9.012835	0.931341	1.06712	Downregulated
**8144279**	-0.09796	3.88E-10	2.18E-08	7.595875	8.129562	0.934352	1.06712	Downregulated
**8033190**	-0.13165	3.90E-10	2.18E-08	8.228967	9.015207	0.912787	1.06712	Downregulated
**8118209**	-0.16975	4.24E-10	2.32E-08	5.99454	6.743056	0.888994	1.06712	Downregulated
**8178043**	-0.16975	4.24E-10	2.32E-08	5.99454	6.743056	0.888994	1.06712	Downregulated
**8179291**	-0.16975	4.24E-10	2.32E-08	5.99454	6.743056	0.888994	1.06712	Downregulated
**7928695**	-0.12654	4.53E-10	2.44E-08	8.183121	8.93328	0.916027	1.06712	Downregulated

**8035842**	-0.12398	4.57E-10	2.45E-08	9.052896	9.865259	0.917654	1.06712	Downregulated
**7923991**	-0.10929	5.29E-10	2.79E-08	7.953572	8.579516	0.927042	1.06712	Downregulated
**7932227**	-0.09819	5.32E-10	2.80E-08	7.43341	7.956923	0.934207	1.06712	Downregulated
**7937892**	-0.10285	5.96E-10	3.08E-08	8.362306	8.980196	0.931194	1.06712	Downregulated
**8014025**	-0.43631	6.05E-10	3.12E-08	7.990116	10.81176	0.739021	1.06712	Downregulated
**7944418**	-0.11338	6.29E-10	3.21E-08	8.497809	9.192577	0.924421	1.06712	Downregulated
**8009145**	-0.1825	6.29E-10	3.21E-08	6.250912	7.093849	0.881173	1.06712	Downregulated
**8100109**	-0.25364	6.36E-10	3.24E-08	5.940058	7.0818	0.838778	1.06712	Downregulated
**8101675**	-0.45452	6.43E-10	3.27E-08	7.49214	10.26666	0.729754	1.06712	Downregulated
**8062728**	-0.17698	7.50E-10	3.72E-08	8.189602	9.258488	0.884551	1.06712	Downregulated
**8132031**	-0.13908	7.53E-10	3.73E-08	9.618289	10.59172	0.908095	1.06712	Downregulated
**7997332**	-0.12496	7.83E-10	3.85E-08	7.771257	8.474379	0.91703	1.06712	Downregulated
**7960117**	-0.13631	7.84E-10	3.85E-08	9.511062	10.45348	0.909846	1.06712	Downregulated
**8140739**	-0.11909	7.89E-10	3.87E-08	6.053926	6.574854	0.92077	1.06712	Downregulated
**7903119**	-0.103	7.93E-10	3.89E-08	9.048772	9.7184	0.931097	1.06712	Downregulated
**8084917**	-0.15989	8.01E-10	3.92E-08	6.945031	7.759021	0.895091	1.06712	Downregulated
**8152369**	-0.10245	8.43E-10	4.08E-08	6.208105	6.664997	0.931449	1.06712	Downregulated

**8117304**	-0.3033	8.67E-10	4.19E-08	7.730878	9.53964	0.810395	1.06712	Downregulated
**7907297**	-0.23749	8.94E-10	4.27E-08	7.069709	8.334742	0.848222	1.06712	Downregulated
**8159385**	-0.10004	9.01E-10	4.30E-08	6.395453	6.854678	0.933006	1.06712	Downregulated
**8099633**	-0.21934	9.14E-10	4.35E-08	7.787379	9.066092	0.858957	1.06712	Downregulated
**7915385**	-0.15118	9.43E-10	4.45E-08	7.413793	8.232833	0.900515	1.06712	Downregulated
**8160663**	-0.14856	9.66E-10	4.53E-08	6.946418	7.69984	0.902151	1.06712	Downregulated
**8145097**	-0.09507	9.66E-10	4.53E-08	4.914518	5.249277	0.936228	1.06712	Downregulated
**8086503**	-0.10256	9.96E-10	4.63E-08	8.622255	9.257545	0.931376	1.06712	Downregulated
**8107859**	-0.11345	1.02E-09	4.74E-08	6.168866	6.673557	0.924375	1.06712	Downregulated
**7960381**	-0.11478	1.03E-09	4.77E-08	6.627102	7.17591	0.923521	1.06712	Downregulated
**7901336**	-0.13902	1.07E-09	4.90E-08	9.859348	10.85672	0.908133	1.06712	Downregulated
**8047443**	-0.14056	1.08E-09	4.94E-08	9.569937	10.54928	0.907165	1.06712	Downregulated
**7915718**	-0.13154	1.08E-09	4.95E-08	7.824379	8.571327	0.912855	1.06712	Downregulated
**8109194**	-0.33846	1.11E-09	5.02E-08	8.832094	11.16739	0.790882	1.06712	Downregulated
**8152053**	-0.09381	1.16E-09	5.25E-08	7.006376	7.477117	0.937042	1.06712	Downregulated
**8059081**	-0.29639	1.27E-09	5.61E-08	6.920638	8.499001	0.814288	1.06712	Downregulated
**8018558**	-0.14678	1.38E-09	5.99E-08	9.755274	10.80005	0.903262	1.06712	Downregulated

**8007043**	-0.15232	1.46E-09	6.33E-08	8.993457	9.994893	0.899805	1.06712	Downregulated
**7917954**	-0.13101	1.61E-09	6.88E-08	7.90389	8.655224	0.913193	1.06712	Downregulated
**8161558**	-0.17134	1.66E-09	7.06E-08	6.112803	6.883646	0.888018	1.06712	Downregulated
**8062339**	-0.10421	1.68E-09	7.11E-08	8.269026	8.888428	0.930314	1.06712	Downregulated
**7952249**	-0.19622	1.78E-09	7.47E-08	7.919732	9.073562	0.872836	1.06712	Downregulated
**8147516**	-0.18151	1.83E-09	7.66E-08	8.165665	9.260453	0.881778	1.06712	Downregulated
**8167603**	-0.10868	1.97E-09	8.12E-08	7.104904	7.660777	0.927439	1.06712	Downregulated
**7919055**	-0.37022	2.00E-09	8.23E-08	8.654906	11.1869	0.773665	1.06712	Downregulated
**7964907**	-0.19963	2.05E-09	8.41E-08	7.128816	8.186768	0.870773	1.06712	Downregulated
**8162531**	-0.0947	2.06E-09	8.41E-08	12.13056	12.9535	0.93647	1.06712	Downregulated
**8121794**	-0.13678	2.10E-09	8.58E-08	9.427414	10.36496	0.909547	1.06712	Downregulated
**8115584**	-0.09916	2.29E-09	9.26E-08	7.738418	8.289005	0.933576	1.06712	Downregulated
**8114006**	-0.21601	2.29E-09	9.26E-08	6.151446	7.145004	0.860944	1.06712	Downregulated
**7931863**	-0.14076	2.32E-09	9.35E-08	7.825845	8.62788	0.907041	1.06712	Downregulated
**7997158**	-0.12147	2.33E-09	9.38E-08	8.334792	9.066931	0.919252	1.06712	Downregulated
**8045587**	-0.16104	2.44E-09	9.76E-08	7.641853	8.544306	0.89438	1.06712	Downregulated
**7910111**	-0.10501	2.69E-09	1.06E-07	7.821344	8.411846	0.929801	1.06712	Downregulated

**7992396**	-0.14113	3.07E-09	1.19E-07	7.294784	8.044464	0.906808	1.06712	Downregulated
**8052956**	-0.12298	3.08E-09	1.19E-07	8.827997	9.613505	0.918291	1.06712	Downregulated
**7956648**	-0.10562	3.16E-09	1.21E-07	7.276604	7.829304	0.929406	1.06712	Downregulated
**8111474**	-0.18087	3.36E-09	1.28E-07	5.514762	6.25137	0.882169	1.06712	Downregulated
**8113403**	-0.09868	3.45E-09	1.31E-07	5.657137	6.057645	0.933884	1.06712	Downregulated
**8166157**	-0.11757	3.45E-09	1.31E-07	5.361568	5.8168	0.921739	1.06712	Downregulated
**7985130**	-0.11642	3.50E-09	1.32E-07	5.511573	5.974781	0.922473	1.06712	Downregulated
**7999079**	-0.10507	3.59E-09	1.35E-07	8.102693	8.714817	0.92976	1.06712	Downregulated
**7959220**	-0.12506	3.61E-09	1.35E-07	9.010529	9.826468	0.916965	1.06712	Downregulated
**8158513**	-0.09496	3.63E-09	1.36E-07	8.211205	8.769879	0.936296	1.06712	Downregulated
**8149097**	-0.16687	3.71E-09	1.38E-07	4.970608	5.5801	0.890774	1.06712	Downregulated
**8037032**	-0.1056	3.72E-09	1.39E-07	6.73063	7.241768	0.929418	1.06712	Downregulated
**7895488**	-0.13228	3.74E-09	1.39E-07	5.996384	6.572163	0.912391	1.06712	Downregulated
**7931852**	-0.25774	3.76E-09	1.40E-07	7.015157	8.387344	0.836398	1.06712	Downregulated
**8117987**	-0.1001	3.79E-09	1.41E-07	8.175052	8.762411	0.932968	1.06712	Downregulated
**7989596**	-0.1068	3.98E-09	1.46E-07	8.401895	9.047442	0.928649	1.06712	Downregulated
**7953303**	-0.15454	4.10E-09	1.49E-07	7.881773	8.772945	0.898418	1.06712	Downregulated

**7980680**	-0.1033	4.20E-09	1.52E-07	9.262452	9.950011	0.930899	1.06712	Downregulated
**7990400**	-0.09687	4.26E-09	1.53E-07	7.822673	8.365957	0.93506	1.06712	Downregulated
**8064967**	-0.10901	4.30E-09	1.54E-07	6.461366	6.968482	0.927227	1.06712	Downregulated
**8168163**	-0.2396	4.35E-09	1.55E-07	6.783369	8.008914	0.846977	1.06712	Downregulated
**7904414**	-0.30187	4.38E-09	1.56E-07	5.864732	7.229673	0.811203	1.06712	Downregulated
**8044700**	-0.23118	5.17E-09	1.81E-07	5.156416	6.052559	0.85194	1.06712	Downregulated
**8053406**	-0.1123	5.74E-09	1.98E-07	10.25868	11.08914	0.92511	1.06712	Downregulated
**7987454**	-0.10925	5.76E-09	1.98E-07	7.41339	7.9966	0.927068	1.06712	Downregulated
**8018251**	-0.11859	6.10E-09	2.08E-07	6.787772	7.369289	0.921089	1.06712	Downregulated
**7924342**	-0.27948	6.28E-09	2.13E-07	7.655184	9.291558	0.823886	1.06712	Downregulated
**8140782**	-0.33125	6.44E-09	2.17E-07	8.176787	10.28721	0.79485	1.06712	Downregulated
**7988852**	-0.11893	6.44E-09	2.17E-07	6.514905	7.074712	0.920872	1.06712	Downregulated
**7949971**	-0.10464	6.46E-09	2.18E-07	9.484693	10.19818	0.930038	1.06712	Downregulated
**8036304**	-0.11072	6.56E-09	2.20E-07	5.231754	5.64906	0.926128	1.06712	Downregulated
**7904429**	-0.2267	6.57E-09	2.20E-07	6.351094	7.431768	0.854587	1.06712	Downregulated
**8100026**	-0.09407	7.14E-09	2.38E-07	8.241948	8.797284	0.936874	1.06712	Downregulated
**8025978**	-0.107	7.15E-09	2.38E-07	6.114809	6.58558	0.928515	1.06712	Downregulated

**8072436**	-0.20564	7.30E-09	2.42E-07	7.451849	8.593483	0.867151	1.06712	Downregulated
**7915404**	-0.28558	7.47E-09	2.48E-07	10.18831	12.41857	0.820409	1.06712	Downregulated
**8111417**	-0.0987	7.54E-09	2.49E-07	5.048612	5.406114	0.933871	1.06712	Downregulated
**8003060**	-0.13615	7.63E-09	2.52E-07	7.020348	7.715117	0.909947	1.06712	Downregulated
**8102352**	-0.10045	8.13E-09	2.64E-07	5.974144	6.404946	0.932739	1.06712	Downregulated
**7966839**	-0.14767	8.44E-09	2.73E-07	8.053397	8.921354	0.90271	1.06712	Downregulated
**8038989**	-0.09528	8.48E-09	2.74E-07	6.491993	6.935219	0.936091	1.06712	Downregulated
**7898249**	-0.16415	8.51E-09	2.75E-07	7.248181	8.121655	0.892451	1.06712	Downregulated
**7923753**	-0.10396	8.56E-09	2.76E-07	8.111087	8.717143	0.930475	1.06712	Downregulated
**8088866**	-0.23471	8.60E-09	2.77E-07	5.624165	6.617805	0.849854	1.06712	Downregulated
**7989951**	-0.16157	8.61E-09	2.77E-07	5.100181	5.704574	0.894051	1.06712	Downregulated
**8151559**	-0.22912	8.69E-09	2.79E-07	7.765753	9.102369	0.853157	1.06712	Downregulated
**8177851**	-0.11163	9.33E-09	2.95E-07	7.980757	8.622825	0.925539	1.06712	Downregulated
**8179167**	-0.11163	9.33E-09	2.95E-07	7.980757	8.622825	0.925539	1.06712	Downregulated
**7904431**	-0.11195	9.48E-09	2.99E-07	5.193931	5.613008	0.925338	1.06712	Downregulated
**8109350**	-0.19763	1.12E-08	3.47E-07	8.586902	9.847587	0.87198	1.06712	Downregulated
**8038919**	-0.10311	1.21E-08	3.71E-07	6.656404	7.149573	0.931021	1.06712	Downregulated

**7977409**	-0.10252	1.27E-08	3.86E-07	7.927913	8.511768	0.931406	1.06712	Downregulated
**7946180**	-0.09576	1.30E-08	3.95E-07	7.379222	7.885628	0.935781	1.06712	Downregulated
**8087419**	-0.0978	1.30E-08	3.95E-07	7.415482	7.935614	0.934456	1.06712	Downregulated
**8083839**	-0.14254	1.30E-08	3.95E-07	8.357985	9.225965	0.90592	1.06712	Downregulated
**8075401**	-0.16025	1.32E-08	4.00E-07	7.702891	8.607828	0.894871	1.06712	Downregulated
**8135847**	-0.10011	1.33E-08	4.00E-07	4.383907	4.698927	0.932959	1.06712	Downregulated
**7947947**	-0.13085	1.37E-08	4.10E-07	5.398728	5.911282	0.913292	1.06712	Downregulated
**8084219**	-0.10307	1.37E-08	4.10E-07	8.39033	9.01172	0.931046	1.06712	Downregulated
**7903407**	-0.14824	1.40E-08	4.19E-07	6.154337	6.820349	0.902349	1.06712	Downregulated
**8163116**	-0.12423	1.46E-08	4.33E-07	7.976519	8.693833	0.917492	1.06712	Downregulated
**8110032**	-0.10191	1.47E-08	4.35E-07	7.78965	8.359793	0.931799	1.06712	Downregulated
**7996569**	-0.21056	1.51E-08	4.45E-07	8.659426	10.02015	0.864202	1.06712	Downregulated
**8161610**	-0.40444	1.55E-08	4.54E-07	5.820475	7.703816	0.755531	1.06712	Downregulated
**7926708**	-0.10289	1.65E-08	4.79E-07	5.845479	6.27759	0.931166	1.06712	Downregulated
**8047097**	-0.12403	1.68E-08	4.86E-07	8.755574	9.541628	0.917619	1.06712	Downregulated
**8138728**	-0.10095	1.71E-08	4.92E-07	6.098924	6.540959	0.93242	1.06712	Downregulated
**8027312**	-0.14882	1.73E-08	4.95E-07	6.359741	7.050814	0.901987	1.06712	Downregulated

**8099696**	-0.12429	1.94E-08	5.47E-07	7.636334	8.323356	0.917458	1.06712	Downregulated
**8079060**	-0.17346	1.99E-08	5.57E-07	8.862722	9.995021	0.886714	1.06712	Downregulated
**7913776**	-0.10716	2.03E-08	5.67E-07	7.479395	8.056091	0.928415	1.06712	Downregulated
**7955441**	-0.1209	2.05E-08	5.73E-07	8.054576	8.758654	0.919613	1.06712	Downregulated
**7934883**	-0.09513	2.29E-08	6.29E-07	5.097013	5.444439	0.936187	1.06712	Downregulated
**8101648**	-0.10746	2.31E-08	6.34E-07	10.80522	11.64077	0.928223	1.06712	Downregulated
**8126214**	-0.11331	2.39E-08	6.52E-07	6.734927	7.285243	0.924461	1.06712	Downregulated
**8038904**	-0.09535	2.53E-08	6.84E-07	6.485039	6.928108	0.936048	1.06712	Downregulated
**8049957**	-0.1615	2.60E-08	7.02E-07	5.682764	6.355873	0.894097	1.06712	Downregulated
**8157233**	-0.10459	2.62E-08	7.06E-07	7.955029	8.553174	0.930068	1.06712	Downregulated
**7941401**	-0.10953	2.82E-08	7.54E-07	8.189936	8.835911	0.926892	1.06712	Downregulated
**7902023**	-0.12253	2.83E-08	7.54E-07	9.352501	10.18151	0.918577	1.06712	Downregulated
**8052742**	-0.11877	2.89E-08	7.65E-07	5.442705	5.909746	0.920971	1.06712	Downregulated
**7943795**	-0.09569	2.92E-08	7.72E-07	7.358411	7.863038	0.935823	1.06712	Downregulated
**8044882**	-0.09531	3.00E-08	7.91E-07	7.579862	8.097528	0.936071	1.06712	Downregulated
**7925250**	-0.13376	3.01E-08	7.92E-07	6.646925	7.292653	0.911455	1.06712	Downregulated
**7916412**	-0.23916	3.14E-08	8.20E-07	6.853724	8.089481	0.847239	1.06712	Downregulated

**7933772**	-0.11825	3.23E-08	8.39E-07	7.322655	7.948112	0.921307	1.06712	Downregulated
**7934945**	-0.10605	3.32E-08	8.60E-07	7.955735	8.562601	0.929126	1.06712	Downregulated
**8163149**	-0.10631	3.36E-08	8.67E-07	8.093867	8.712834	0.928959	1.06712	Downregulated
**7915500**	-0.14341	3.41E-08	8.80E-07	9.04122	9.986137	0.905377	1.06712	Downregulated
**8101207**	-0.09602	3.44E-08	8.86E-07	6.724222	7.186974	0.935612	1.06712	Downregulated
**8116835**	-0.21117	3.47E-08	8.92E-07	6.046006	6.999012	0.863837	1.06712	Downregulated
**8140371**	-0.12042	3.68E-08	9.39E-07	8.442658	9.177597	0.91992	1.06712	Downregulated
**7913727**	-0.10576	3.83E-08	9.72E-07	6.301751	6.781088	0.929313	1.06712	Downregulated
**8101284**	-0.45985	3.93E-08	9.93E-07	6.000202	8.252659	0.727063	1.06712	Downregulated
**8131550**	-0.24547	4.11E-08	1.03E-06	7.980942	9.461249	0.84354	1.06712	Downregulated
**8164967**	-0.10057	4.18E-08	1.05E-06	8.050077	8.631248	0.932667	1.06712	Downregulated
**8086185**	-0.14028	4.26E-08	1.07E-06	7.749347	8.540683	0.907345	1.06712	Downregulated
**7912937**	-0.26441	4.38E-08	1.09E-06	8.669197	10.413	0.832536	1.06712	Downregulated
**8011375**	-0.09793	4.50E-08	1.12E-06	7.178967	7.683187	0.934374	1.06712	Downregulated
**8160504**	-0.22275	4.88E-08	1.20E-06	8.295416	9.680374	0.856931	1.06712	Downregulated
**8101780**	-0.20454	4.93E-08	1.21E-06	6.075207	7.000564	0.867817	1.06712	Downregulated
**7919314**	-0.21033	5.03E-08	1.23E-06	8.451415	9.777898	0.864339	1.06712	Downregulated

**8022106**	-0.11737	5.32E-08	1.29E-06	6.972804	7.563811	0.921864	1.06712	Downregulated
**8027304**	-0.10243	5.51E-08	1.32E-06	6.549778	7.031706	0.931464	1.06712	Downregulated
**8046186**	-0.09604	5.94E-08	1.41E-06	6.316023	6.7508	0.935596	1.06712	Downregulated
**7966448**	-0.14257	5.96E-08	1.42E-06	6.980873	7.705969	0.905905	1.06712	Downregulated
**7967107**	-0.21452	6.89E-08	1.62E-06	7.883358	9.147209	0.861832	1.06712	Downregulated
**8126153**	-0.16052	6.89E-08	1.62E-06	8.054291	9.002172	0.894705	1.06712	Downregulated
**8116316**	-0.097	7.50E-08	1.74E-06	8.937467	9.559056	0.934974	1.06712	Downregulated
**7936734**	-0.1619	7.82E-08	1.80E-06	6.936636	7.760446	0.893845	1.06712	Downregulated
**7994123**	-0.26889	8.31E-08	1.90E-06	8.417586	10.14219	0.829958	1.06712	Downregulated
**7964577**	-0.09529	9.32E-08	2.10E-06	6.272776	6.701092	0.936083	1.06712	Downregulated
**8144712**	-0.09626	9.38E-08	2.11E-06	4.791623	5.122234	0.935456	1.06712	Downregulated
**8045974**	-0.14532	9.49E-08	2.13E-06	5.34262	5.908805	0.90418	1.06712	Downregulated
**8171708**	-0.09634	9.56E-08	2.14E-06	6.437382	6.881925	0.935404	1.06712	Downregulated
**7982927**	-0.09506	9.98E-08	2.22E-06	7.457169	7.965076	0.936233	1.06712	Downregulated
**8016457**	-0.15748	1.00E-07	2.23E-06	7.028148	7.838754	0.89659	1.06712	Downregulated
**8092473**	-0.11992	1.02E-07	2.26E-06	8.507096	9.244435	0.92024	1.06712	Downregulated
**7984588**	-0.1176	1.06E-07	2.34E-06	7.335471	7.958483	0.921717	1.06712	Downregulated

**8078784**	-0.11008	1.08E-07	2.38E-06	7.830754	8.451659	0.926535	1.06712	Downregulated
**7946454**	-0.15749	1.12E-07	2.44E-06	6.710952	7.485009	0.896586	1.06712	Downregulated
**8056877**	-0.10223	1.12E-07	2.46E-06	6.006523	6.447607	0.931589	1.06712	Downregulated
**7943162**	-0.09865	1.19E-07	2.57E-06	8.863567	9.490841	0.933907	1.06712	Downregulated
**8143629**	-0.09905	1.20E-07	2.60E-06	7.519209	8.053595	0.933646	1.06712	Downregulated
**8049349**	-0.21296	1.21E-07	2.60E-06	7.534265	8.732714	0.862763	1.06712	Downregulated
**8130645**	-0.10561	1.22E-07	2.62E-06	6.333363	6.814382	0.929411	1.06712	Downregulated
**8030831**	-0.0978	1.23E-07	2.64E-06	6.490728	6.946	0.934456	1.06712	Downregulated
**8116921**	-0.13335	1.46E-07	3.06E-06	7.694643	8.439793	0.91171	1.06712	Downregulated
**8098654**	-0.18297	1.46E-07	3.06E-06	5.483293	6.224721	0.88089	1.06712	Downregulated
**8063729**	-0.14714	1.48E-07	3.09E-06	8.671654	9.602742	0.903039	1.06712	Downregulated
**8094938**	-0.12528	1.50E-07	3.14E-06	8.503701	9.275152	0.916826	1.06712	Downregulated
**8165538**	-0.11234	1.53E-07	3.19E-06	8.5768	9.271342	0.925087	1.06712	Downregulated
**8086467**	-0.10097	1.54E-07	3.21E-06	9.388816	10.06943	0.932408	1.06712	Downregulated
**8098637**	-0.10126	1.60E-07	3.29E-06	8.998683	9.652948	0.932221	1.06712	Downregulated
**8088142**	-0.13144	1.67E-07	3.42E-06	8.322783	9.116654	0.912921	1.06712	Downregulated
**7930194**	-0.11236	1.78E-07	3.62E-06	7.772263	8.401775	0.925074	1.06712	Downregulated

**7920744**	-0.11028	1.86E-07	3.74E-06	6.525308	7.043684	0.926406	1.06712	Downregulated
**7950082**	-0.0983	2.01E-07	3.98E-06	6.94598	7.43575	0.934133	1.06712	Downregulated
**8166925**	-0.17675	2.04E-07	4.04E-06	8.959803	10.12758	0.884693	1.06712	Downregulated
**8006655**	-0.20286	2.06E-07	4.07E-06	10.07794	11.59946	0.868828	1.06712	Downregulated
**7965964**	-0.09513	2.11E-07	4.15E-06	9.598833	10.25307	0.936191	1.06712	Downregulated
**8139680**	-0.13663	2.13E-07	4.19E-06	7.774341	8.546615	0.90964	1.06712	Downregulated
**7944478**	-0.19609	2.20E-07	4.31E-06	7.402521	8.480267	0.872911	1.06712	Downregulated
**8056151**	-0.15712	2.35E-07	4.58E-06	7.188894	8.016013	0.896817	1.06712	Downregulated
**8110971**	-0.1442	2.35E-07	4.58E-06	9.454137	10.4479	0.904884	1.06712	Downregulated
**7958273**	-0.12853	2.36E-07	4.58E-06	5.363605	5.863381	0.914763	1.06712	Downregulated
**7960730**	-0.10583	2.46E-07	4.74E-06	10.9791	11.81477	0.92927	1.06712	Downregulated
**8107632**	-0.10781	2.68E-07	5.08E-06	6.528896	7.035471	0.927997	1.06712	Downregulated
**8166784**	-0.11946	2.70E-07	5.10E-06	9.716221	10.55498	0.920534	1.06712	Downregulated
**8123658**	-0.10486	2.70E-07	5.11E-06	8.544929	9.189101	0.929898	1.06712	Downregulated
**7911767**	-0.11406	2.77E-07	5.20E-06	6.006217	6.500324	0.923987	1.06712	Downregulated
**7923700**	-0.10735	2.83E-07	5.29E-06	6.619459	7.130779	0.928294	1.06712	Downregulated
**8004922**	-0.11384	2.84E-07	5.31E-06	6.077976	6.577023	0.924123	1.06712	Downregulated

**8009685**	-0.11789	3.02E-07	5.61E-06	10.51824	11.41387	0.921531	1.06712	Downregulated
**8028991**	-0.14887	3.11E-07	5.76E-06	9.486048	10.51718	0.901958	1.06712	Downregulated
**7919669**	-0.1289	3.17E-07	5.85E-06	8.993462	9.833987	0.914529	1.06712	Downregulated
**8044813**	-0.16824	3.20E-07	5.89E-06	9.218304	10.3585	0.889926	1.06712	Downregulated
**8095402**	-0.4104	3.76E-07	6.76E-06	6.229502	8.279344	0.752415	1.06712	Downregulated
**8063590**	-0.33477	3.78E-07	6.78E-06	8.500649	10.7208	0.792912	1.06712	Downregulated
**7978970**	-0.09698	4.04E-07	7.19E-06	6.049386	6.470022	0.934987	1.06712	Downregulated
**8044499**	-0.10308	4.11E-07	7.28E-06	8.707253	9.35217	0.931041	1.06712	Downregulated
**7894864**	-0.13152	4.14E-07	7.32E-06	6.644175	7.278365	0.912866	1.06712	Downregulated
**8004534**	-0.1735	4.39E-07	7.65E-06	6.046943	6.819671	0.886691	1.06712	Downregulated
**8083848**	-0.17394	4.39E-07	7.65E-06	4.984631	5.623354	0.886416	1.06712	Downregulated
**8013660**	-0.17189	4.41E-07	7.68E-06	7.943792	8.948952	0.887678	1.06712	Downregulated
**8053325**	-0.09425	4.53E-07	7.85E-06	4.739829	5.059819	0.936759	1.06712	Downregulated
**8058498**	-0.10733	4.59E-07	7.94E-06	9.46403	10.19497	0.928304	1.06712	Downregulated
**8164087**	-0.09796	4.61E-07	7.96E-06	6.738308	7.211723	0.934355	1.06712	Downregulated
**8122744**	-0.16788	5.15E-07	8.74E-06	7.059918	7.931145	0.890151	1.06712	Downregulated
**7965156**	-0.16725	5.20E-07	8.80E-06	5.675124	6.372675	0.89054	1.06712	Downregulated

**8059067**	-0.1112	5.27E-07	8.89E-06	8.309769	8.975578	0.92582	1.06712	Downregulated
**8003263**	-0.11381	5.45E-07	9.15E-06	7.922144	8.57239	0.924147	1.06712	Downregulated
**7956009**	-0.17252	5.54E-07	9.23E-06	8.791493	9.908251	0.88729	1.06712	Downregulated
**8158224**	-0.10719	5.78E-07	9.58E-06	8.983072	9.675938	0.928393	1.06712	Downregulated
**8111490**	-0.2116	6.09E-07	1.00E-05	7.460937	8.639534	0.863581	1.06712	Downregulated
**8084929**	-0.29177	6.35E-07	1.04E-05	7.58194	9.281363	0.816899	1.06712	Downregulated
**8141066**	-0.19344	6.49E-07	1.06E-05	6.758272	7.727973	0.874521	1.06712	Downregulated
**7988426**	-0.15524	6.64E-07	1.08E-05	8.48541	9.449383	0.897986	1.06712	Downregulated
**7958000**	-0.09959	7.03E-07	1.13E-05	9.832274	10.53499	0.933297	1.06712	Downregulated
**8002283**	-0.16901	7.09E-07	1.14E-05	5.912871	6.647777	0.889451	1.06712	Downregulated
**7901229**	-0.09718	7.32E-07	1.17E-05	7.529189	8.05381	0.93486	1.06712	Downregulated
**8053030**	-0.3087	7.53E-07	1.20E-05	6.994728	8.663631	0.807367	1.06712	Downregulated
**8164535**	-0.10898	7.64E-07	1.21E-05	9.141265	9.858569	0.927241	1.06712	Downregulated
**7942489**	-0.13424	7.80E-07	1.23E-05	5.405304	5.93239	0.911151	1.06712	Downregulated
**8051275**	-0.2659	8.00E-07	1.26E-05	7.371032	8.862822	0.83168	1.06712	Downregulated
**8081758**	-0.18141	8.06E-07	1.27E-05	7.238688	8.208636	0.881838	1.06712	Downregulated
**7942774**	-0.2169	8.11E-07	1.27E-05	7.13513	8.292664	0.860415	1.06712	Downregulated

**8173825**	-0.17502	8.87E-07	1.37E-05	6.418946	7.246854	0.885756	1.06712	Downregulated
**8111864**	-0.0963	8.92E-07	1.38E-05	4.561681	4.876565	0.935429	1.06712	Downregulated
**7929383**	-0.20461	8.93E-07	1.38E-05	7.734858	8.913453	0.867773	1.06712	Downregulated
**8138749**	-0.14351	9.03E-07	1.39E-05	7.749526	8.560023	0.905316	1.06712	Downregulated
**8173245**	-0.10788	9.19E-07	1.41E-05	6.329365	6.82081	0.927949	1.06712	Downregulated
**8093166**	-0.20834	9.21E-07	1.41E-05	7.599537	8.780197	0.865532	1.06712	Downregulated
**7915682**	-0.11365	9.26E-07	1.42E-05	8.207374	8.880049	0.924249	1.06712	Downregulated
**8109303**	-0.18822	9.36E-07	1.43E-05	4.541808	5.174739	0.877688	1.06712	Downregulated
**8138647**	-0.1079	9.40E-07	1.44E-05	6.974048	7.515648	0.927937	1.06712	Downregulated
**7949344**	-0.31555	9.81E-07	1.49E-05	8.024267	9.986062	0.803547	1.06712	Downregulated
**7945774**	-0.12078	9.94E-07	1.51E-05	6.750324	7.339757	0.919693	1.06712	Downregulated
**8166723**	-0.10414	1.01E-06	1.53E-05	8.464162	9.097735	0.930359	1.06712	Downregulated
**8026468**	-0.18504	1.04E-06	1.57E-05	7.803573	8.871508	0.879622	1.06712	Downregulated
**7988212**	-0.11136	1.05E-06	1.57E-05	6.735638	7.276131	0.925717	1.06712	Downregulated
**7933640**	-0.16746	1.05E-06	1.57E-05	8.937808	10.03787	0.890409	1.06712	Downregulated
**8085122**	-0.13294	1.11E-06	1.66E-05	6.853615	7.515148	0.911973	1.06712	Downregulated
**8161371**	-0.2102	1.18E-06	1.74E-05	7.386516	8.545051	0.86442	1.06712	Downregulated

**8108078**	-0.10171	1.22E-06	1.80E-05	5.357178	5.748483	0.931929	1.06712	Downregulated
**8085797**	-0.15307	1.24E-06	1.81E-05	6.941794	7.718785	0.899338	1.06712	Downregulated
**8024557**	-0.10059	1.25E-06	1.82E-05	10.75372	11.53027	0.932651	1.06712	Downregulated
**7898112**	-0.09955	1.31E-06	1.90E-05	5.524249	5.918884	0.933326	1.06712	Downregulated
**8100760**	-0.46379	1.34E-06	1.94E-05	6.83112	9.42118	0.725081	1.06712	Downregulated
**8135341**	-0.10949	1.45E-06	2.07E-05	5.304731	5.722987	0.926916	1.06712	Downregulated
**7909862**	-0.13329	1.52E-06	2.15E-05	8.459651	9.278476	0.91175	1.06712	Downregulated
**7983270**	-0.09559	1.54E-06	2.18E-05	4.903079	5.238949	0.93589	1.06712	Downregulated
**8072636**	-0.17506	1.66E-06	2.33E-05	7.621516	8.604798	0.885729	1.06712	Downregulated
**8101637**	-0.09546	1.71E-06	2.38E-05	4.775081	5.101735	0.935972	1.06712	Downregulated
**8132013**	-0.17457	1.72E-06	2.40E-05	8.61306	9.720941	0.886031	1.06712	Downregulated
**7994252**	-0.38858	1.74E-06	2.42E-05	6.924896	9.065395	0.763882	1.06712	Downregulated
**8139881**	-0.09443	1.76E-06	2.44E-05	6.771663	7.229726	0.936642	1.06712	Downregulated
**8154135**	-0.19287	1.78E-06	2.47E-05	8.777385	10.03286	0.874863	1.06712	Downregulated
**8096116**	-0.13217	1.93E-06	2.64E-05	8.589905	9.414016	0.912459	1.06712	Downregulated
**8138822**	-0.11444	1.95E-06	2.67E-05	5.306264	5.74434	0.923738	1.06712	Downregulated
**7979804**	-0.13935	1.95E-06	2.67E-05	5.14571	5.667527	0.907929	1.06712	Downregulated

**8054135**	-0.10114	1.96E-06	2.68E-05	9.663603	10.3654	0.932295	1.06712	Downregulated
**7943605**	-0.12395	1.96E-06	2.68E-05	7.797449	8.496967	0.917674	1.06712	Downregulated
**7930454**	-0.1085	1.98E-06	2.70E-05	9.229239	9.950108	0.927552	1.06712	Downregulated
**7980742**	-0.10113	1.98E-06	2.70E-05	4.857705	5.210437	0.932303	1.06712	Downregulated
**7895359**	-0.14655	2.09E-06	2.83E-05	6.279554	6.950972	0.903407	1.06712	Downregulated
**8116635**	-0.15355	2.11E-06	2.84E-05	7.471011	8.310007	0.899038	1.06712	Downregulated
**8018652**	-0.12382	2.14E-06	2.88E-05	8.016667	8.735112	0.917752	1.06712	Downregulated
**8096744**	-0.12078	2.16E-06	2.89E-05	6.410012	6.96973	0.919693	1.06712	Downregulated
**8079117**	-0.11168	2.20E-06	2.94E-05	6.345528	6.856241	0.925511	1.06712	Downregulated
**8062284**	-0.11566	2.26E-06	3.01E-05	5.60121	6.068763	0.922958	1.06712	Downregulated
**8093171**	-0.09509	2.27E-06	3.02E-05	8.051701	8.600255	0.936217	1.06712	Downregulated
**8146687**	-0.12139	2.29E-06	3.04E-05	5.94511	6.466991	0.919301	1.06712	Downregulated
**7900488**	-0.09671	2.31E-06	3.06E-05	5.146068	5.502837	0.935166	1.06712	Downregulated
**7977033**	-0.09699	2.38E-06	3.14E-05	9.158741	9.795632	0.934982	1.06712	Downregulated
**7912659**	-0.10352	2.41E-06	3.18E-05	8.712985	9.361129	0.930762	1.06712	Downregulated
**8055323**	-0.17709	2.50E-06	3.27E-05	5.968406	6.747898	0.884484	1.06712	Downregulated
**7901613**	-0.09481	2.55E-06	3.33E-05	8.506896	9.084718	0.936396	1.06712	Downregulated

**8087907**	-0.11154	2.56E-06	3.34E-05	6.773133	7.317581	0.925597	1.06712	Downregulated
**8128726**	-0.09478	2.56E-06	3.34E-05	5.39443	5.760712	0.936417	1.06712	Downregulated
**7901513**	-0.09891	2.56E-06	3.34E-05	9.019675	9.659716	0.933741	1.06712	Downregulated
**7909628**	-0.22795	2.58E-06	3.36E-05	7.868373	9.215191	0.853848	1.06712	Downregulated
**8059783**	-0.11745	2.59E-06	3.37E-05	7.689547	8.341726	0.921817	1.06712	Downregulated
**8173366**	-0.12594	2.64E-06	3.42E-05	6.352022	6.931422	0.91641	1.06712	Downregulated
**8030954**	-0.09683	2.64E-06	3.42E-05	5.207735	5.569264	0.935085	1.06712	Downregulated
**8103494**	-0.20286	2.65E-06	3.43E-05	4.355947	5.013592	0.868827	1.06712	Downregulated
**8114002**	-0.12371	2.67E-06	3.45E-05	6.028749	6.56851	0.917826	1.06712	Downregulated
**8013022**	-0.12526	2.67E-06	3.45E-05	9.222926	10.05945	0.916842	1.06712	Downregulated
**7986394**	-0.10478	2.73E-06	3.52E-05	8.499573	9.139856	0.929946	1.06712	Downregulated
**8024566**	-0.11769	2.73E-06	3.52E-05	10.27056	11.14352	0.921662	1.06712	Downregulated
**8110980**	-0.13689	2.88E-06	3.69E-05	4.711583	5.18052	0.909481	1.06712	Downregulated
**8078386**	-0.10924	3.08E-06	3.90E-05	8.082111	8.717863	0.927075	1.06712	Downregulated
**8133938**	-0.16452	3.14E-06	3.96E-05	7.316467	8.200264	0.892223	1.06712	Downregulated
**8056491**	-0.15113	3.14E-06	3.96E-05	5.696432	6.325519	0.900548	1.06712	Downregulated
**8025984**	-0.10384	3.24E-06	4.07E-05	5.78577	6.21756	0.930553	1.06712	Downregulated

**7925691**	-0.10558	3.25E-06	4.08E-05	6.719813	7.230005	0.929434	1.06712	Downregulated
**8081925**	-0.1329	3.27E-06	4.10E-05	7.383139	8.095581	0.911996	1.06712	Downregulated
**8113666**	-0.12871	3.29E-06	4.13E-05	9.051362	9.89598	0.91465	1.06712	Downregulated
**8111512**	-0.14196	3.30E-06	4.13E-05	4.630071	5.108817	0.90629	1.06712	Downregulated
**8097829**	-0.09612	3.32E-06	4.16E-05	7.197077	7.692932	0.935544	1.06712	Downregulated
**7915598**	-0.20286	3.38E-06	4.22E-05	7.777624	8.9519	0.868824	1.06712	Downregulated
**7969426**	-0.10737	3.49E-06	4.34E-05	3.887297	4.187649	0.928276	1.06712	Downregulated
**7926403**	-0.23084	3.56E-06	4.42E-05	8.820704	10.35124	0.852139	1.06712	Downregulated
**7926445**	-0.23084	3.56E-06	4.42E-05	8.820704	10.35124	0.852139	1.06712	Downregulated
**8091283**	-0.11221	3.62E-06	4.48E-05	9.102818	9.839108	0.925167	1.06712	Downregulated
**8006906**	-0.09706	3.63E-06	4.48E-05	8.910214	9.530313	0.934934	1.06712	Downregulated
**7956229**	-0.18082	3.68E-06	4.54E-05	9.65938	10.94914	0.882204	1.06712	Downregulated
**7944339**	-0.1187	3.78E-06	4.64E-05	6.500516	7.057989	0.921015	1.06712	Downregulated
**8113577**	-0.1342	3.87E-06	4.75E-05	6.717327	7.372151	0.911176	1.06712	Downregulated
**8121212**	-0.16141	3.94E-06	4.82E-05	7.380663	8.254358	0.894153	1.06712	Downregulated
**8013015**	-0.108	4.09E-06	4.99E-05	8.389191	9.041294	0.927875	1.06712	Downregulated
**8006415**	-0.09496	4.19E-06	5.09E-05	8.456923	9.032272	0.936301	1.06712	Downregulated

**7913787**	-0.10122	4.23E-06	5.13E-05	7.062467	7.575774	0.932244	1.06712	Downregulated
**8044346**	-0.10932	4.46E-06	5.37E-05	8.82824	9.523187	0.927026	1.06712	Downregulated
**7919984**	-0.11907	4.54E-06	5.46E-05	10.56874	11.47805	0.920778	1.06712	Downregulated
**8151496**	-0.13222	4.61E-06	5.52E-05	7.065784	7.743963	0.912425	1.06712	Downregulated
**7919305**	-0.11936	4.63E-06	5.54E-05	8.367749	9.089509	0.920594	1.06712	Downregulated
**7902977**	-0.10877	4.78E-06	5.68E-05	6.11385	6.592633	0.927376	1.06712	Downregulated
**8144866**	-0.15732	5.17E-06	6.07E-05	6.86828	7.659601	0.896689	1.06712	Downregulated
**8041727**	-0.25208	5.23E-06	6.13E-05	7.255203	8.64038	0.839686	1.06712	Downregulated
**7985192**	-0.13374	5.34E-06	6.23E-05	7.15945	7.854881	0.911465	1.06712	Downregulated
**7916616**	-0.18211	5.74E-06	6.62E-05	8.256306	9.367115	0.881414	1.06712	Downregulated
**8087224**	-0.12469	5.81E-06	6.69E-05	8.349228	9.102923	0.917203	1.06712	Downregulated
**7928882**	-0.18109	5.96E-06	6.82E-05	7.93045	8.991101	0.882033	1.06712	Downregulated
**8120088**	-0.19791	6.03E-06	6.89E-05	10.68189	12.25248	0.871814	1.06712	Downregulated
**8153762**	-0.1361	6.12E-06	6.97E-05	8.638602	9.49325	0.909973	1.06712	Downregulated
**8106170**	-0.12031	6.17E-06	7.02E-05	8.435487	9.169105	0.91999	1.06712	Downregulated
**8128837**	-0.16456	6.41E-06	7.26E-05	5.22965	5.861516	0.892201	1.06712	Downregulated
**8035083**	-0.29783	6.56E-06	7.40E-05	7.603515	9.346925	0.813478	1.06712	Downregulated

**7996345**	-0.11571	6.60E-06	7.44E-05	10.42066	11.29087	0.922928	1.06712	Downregulated
**8069795**	-0.4365	6.66E-06	7.50E-05	5.459812	7.388853	0.738926	1.06712	Downregulated
**8035813**	-0.11343	6.79E-06	7.62E-05	6.21217	6.720298	0.924389	1.06712	Downregulated
**7894427**	-0.12752	6.80E-06	7.62E-05	5.897142	6.442115	0.915405	1.06712	Downregulated
**8027519**	-0.15152	7.02E-06	7.81E-05	3.419084	3.79771	0.900301	1.06712	Downregulated
**7935169**	-0.21861	7.04E-06	7.83E-05	4.678907	5.444441	0.859392	1.06712	Downregulated
**8117415**	-0.11622	7.23E-06	8.01E-05	6.419451	6.957987	0.922602	1.06712	Downregulated
**8101862**	-0.21116	7.27E-06	8.05E-05	7.297441	8.447659	0.863842	1.06712	Downregulated
**8052418**	-0.15984	7.56E-06	8.33E-05	6.77981	7.574143	0.895126	1.06712	Downregulated
**8141094**	-0.14078	7.64E-06	8.41E-05	8.829248	9.734284	0.907026	1.06712	Downregulated
**8091861**	-0.20215	7.72E-06	8.47E-05	4.3343	4.986227	0.869254	1.06712	Downregulated
**8039044**	-0.11707	7.76E-06	8.51E-05	5.436389	5.895946	0.922055	1.06712	Downregulated
**8008802**	-0.13343	7.83E-06	8.57E-05	5.949479	6.525981	0.91166	1.06712	Downregulated
**8166511**	-0.09801	7.94E-06	8.68E-05	8.058129	8.624606	0.934319	1.06712	Downregulated
**8145532**	-0.19242	7.99E-06	8.73E-05	7.797523	8.910073	0.875136	1.06712	Downregulated
**8035855**	-0.14148	8.05E-06	8.78E-05	5.124169	5.652126	0.906591	1.06712	Downregulated
**8143461**	-0.10336	8.06E-06	8.79E-05	7.922447	8.510876	0.930861	1.06712	Downregulated

**8043833**	-0.09778	8.22E-06	8.94E-05	3.594648	3.846729	0.934469	1.06712	Downregulated
**7975081**	-0.10523	8.39E-06	9.09E-05	6.186326	6.654409	0.929658	1.06712	Downregulated
**7959234**	-0.10088	8.61E-06	9.29E-05	7.596797	8.146996	0.932466	1.06712	Downregulated
**7992841**	-0.14081	8.66E-06	9.34E-05	5.966182	6.577852	0.907011	1.06712	Downregulated
**8000590**	-0.14417	8.96E-06	9.62E-05	7.023377	7.761518	0.904897	1.06712	Downregulated
**8135544**	-0.16778	8.99E-06	9.64E-05	5.753867	6.46349	0.890211	1.06712	Downregulated
**7975799**	-0.132	9.03E-06	9.66E-05	8.714821	9.549799	0.912566	1.06712	Downregulated
**7895511**	-0.1604	9.10E-06	9.71E-05	6.426277	7.182012	0.894774	1.06712	Downregulated
**8107909**	-0.19035	9.30E-06	9.89E-05	7.011953	8.000905	0.876395	1.06712	Downregulated
**8054517**	-0.104	9.37E-06	9.95E-05	9.874807	10.61291	0.930452	1.06712	Downregulated
**8019486**	-0.1191	9.67E-06	0.000102	9.509892	10.32832	0.920759	1.06712	Downregulated
**8156082**	-0.09542	9.73E-06	0.000103	4.327369	4.623274	0.935997	1.06712	Downregulated
**8035787**	-0.12087	9.79E-06	0.000103	5.137496	5.586445	0.919636	1.06712	Downregulated
**8000582**	-0.2079	9.86E-06	0.000104	8.587309	9.918399	0.865796	1.06712	Downregulated
**8141342**	-0.22611	9.87E-06	0.000104	5.801386	6.78574	0.854938	1.06712	Downregulated
**8166714**	-0.12647	1.00E-05	0.000105	5.696559	6.218476	0.91607	1.06712	Downregulated
**7957819**	-0.15731	1.01E-05	0.000106	5.715182	6.373624	0.896693	1.06712	Downregulated

**8042062**	-0.09599	1.01E-05	0.000106	4.518637	4.829508	0.935631	1.06712	Downregulated
**8077899**	-0.11904	1.08E-05	0.000112	8.680939	9.427612	0.920799	1.06712	Downregulated
**7980477**	-0.09679	1.09E-05	0.000112	5.889906	6.298601	0.935113	1.06712	Downregulated
**8012830**	-0.16853	1.09E-05	0.000113	7.211949	8.105603	0.889749	1.06712	Downregulated
**8103374**	-0.11217	1.11E-05	0.000114	5.914308	6.392507	0.925194	1.06712	Downregulated
**8142628**	-0.51875	1.12E-05	0.000115	5.815131	8.331441	0.697974	1.06712	Downregulated
**8048319**	-0.12799	1.12E-05	0.000115	10.21662	11.16441	0.915106	1.06712	Downregulated
**8013616**	-0.11979	1.12E-05	0.000115	7.781182	8.454855	0.920321	1.06712	Downregulated
**8018038**	-0.1129	1.19E-05	0.000122	7.64001	8.261903	0.924728	1.06712	Downregulated
**8110685**	-0.13884	1.24E-05	0.000125	9.466204	10.42245	0.908251	1.06712	Downregulated
**8169174**	-0.12744	1.26E-05	0.000127	8.298906	9.06533	0.915455	1.06712	Downregulated
**7994216**	-0.23805	1.27E-05	0.000128	6.480943	7.643625	0.847889	1.06712	Downregulated
**8166408**	-0.12301	1.28E-05	0.000129	5.269702	5.738732	0.918269	1.06712	Downregulated
**8080562**	-0.12229	1.29E-05	0.000129	7.094904	7.722506	0.918731	1.06712	Downregulated
**7933982**	-0.22463	1.32E-05	0.000132	8.301197	9.699761	0.855815	1.06712	Downregulated
**8035847**	-0.10404	1.33E-05	0.000133	6.837804	7.349103	0.930427	1.06712	Downregulated
**7933750**	-0.27652	1.36E-05	0.000136	6.265067	7.58868	0.825581	1.06712	Downregulated

**8047036**	-0.09488	1.40E-05	0.00014	4.456524	4.759461	0.936351	1.06712	Downregulated
**7929986**	-0.11866	1.41E-05	0.000141	4.067669	4.416363	0.921045	1.06712	Downregulated
**8016735**	-0.15532	1.47E-05	0.000146	8.343221	9.291613	0.89793	1.06712	Downregulated
**8102065**	-0.16795	1.51E-05	0.000149	6.479822	7.279825	0.890107	1.06712	Downregulated
**8059955**	-0.14457	1.51E-05	0.00015	7.692247	8.502996	0.904651	1.06712	Downregulated
**8067877**	-0.13588	1.62E-05	0.000159	5.708222	6.271999	0.910112	1.06712	Downregulated
**8110606**	-0.16432	1.64E-05	0.000161	9.088255	10.18464	0.892349	1.06712	Downregulated
**7962455**	-0.17283	1.66E-05	0.000163	5.695708	6.420597	0.887099	1.06712	Downregulated
**7905406**	-0.09712	1.70E-05	0.000166	8.418431	9.004646	0.934899	1.06712	Downregulated
**8162652**	-0.10264	1.75E-05	0.000171	5.284942	5.674641	0.931326	1.06712	Downregulated
**8055488**	-0.10862	1.77E-05	0.000172	2.776692	2.993812	0.927477	1.06712	Downregulated
**8072360**	-0.13833	1.85E-05	0.000178	8.918502	9.81594	0.908573	1.06712	Downregulated
**7973850**	-0.09477	1.96E-05	0.000188	5.724616	6.1133	0.93642	1.06712	Downregulated
**7939173**	-0.19165	2.01E-05	0.000191	6.510627	7.435598	0.875602	1.06712	Downregulated
**7925436**	-0.15182	2.05E-05	0.000194	6.153322	6.836162	0.900114	1.06712	Downregulated
**8163328**	-0.19941	2.13E-05	0.0002	7.822128	8.981605	0.870905	1.06712	Downregulated
**8075956**	-0.17181	2.15E-05	0.000202	7.949111	8.954466	0.887726	1.06712	Downregulated

**8002667**	-0.10736	2.17E-05	0.000204	5.832092	6.282641	0.928287	1.06712	Downregulated
**8116537**	-0.16074	2.18E-05	0.000204	9.236181	10.32478	0.894565	1.06712	Downregulated
**7902290**	-0.10208	2.24E-05	0.000209	6.740664	7.234877	0.93169	1.06712	Downregulated
**7914342**	-0.10182	2.29E-05	0.000214	5.552513	5.958556	0.931856	1.06712	Downregulated
**8080212**	-0.09643	2.35E-05	0.000218	7.645498	8.17397	0.935347	1.06712	Downregulated
**8054513**	-0.11109	2.45E-05	0.000226	6.931162	7.485976	0.925886	1.06712	Downregulated
**8022118**	-0.11734	2.49E-05	0.000229	8.581816	9.30899	0.921885	1.06712	Downregulated
**8007550**	-0.10108	2.52E-05	0.000231	8.094945	8.682431	0.932336	1.06712	Downregulated
**8110688**	-0.15401	2.56E-05	0.000234	9.34527	10.39809	0.898749	1.06712	Downregulated
**8136336**	-0.188	2.66E-05	0.000242	9.038194	10.29618	0.87782	1.06712	Downregulated
**8088636**	-0.11633	2.71E-05	0.000244	6.098177	6.610246	0.922534	1.06712	Downregulated
**8064900**	-0.10273	2.72E-05	0.000245	5.596487	6.009523	0.93127	1.06712	Downregulated
**8154100**	-0.14539	2.73E-05	0.000246	5.860934	6.482386	0.904132	1.06712	Downregulated
**7945475**	-0.13603	2.74E-05	0.000247	10.09347	11.09149	0.91002	1.06712	Downregulated
**7994615**	-0.20765	2.78E-05	0.00025	9.56695	11.04799	0.865945	1.06712	Downregulated
**7908597**	-0.09479	2.85E-05	0.000255	9.53197	10.17929	0.936408	1.06712	Downregulated
**8056583**	-0.09704	2.88E-05	0.000256	4.898642	5.23947	0.93495	1.06712	Downregulated

**8141872**	-0.09927	3.01E-05	0.000266	8.403771	9.002372	0.933506	1.06712	Downregulated
**8129888**	-0.11264	3.15E-05	0.000278	9.175503	9.920579	0.924896	1.06712	Downregulated
**7895634**	-0.11	3.19E-05	0.000281	4.790715	5.17029	0.926585	1.06712	Downregulated
**7990555**	-0.14703	3.32E-05	0.00029	4.693677	5.19724	0.90311	1.06712	Downregulated
**8003814**	-0.31201	3.41E-05	0.000297	5.288858	6.565794	0.805517	1.06712	Downregulated
**7931571**	-0.16287	3.45E-05	0.000299	8.4691	9.481273	0.893245	1.06712	Downregulated
**7916789**	-0.16345	3.56E-05	0.000308	5.743747	6.43278	0.892887	1.06712	Downregulated
**8099326**	-0.12993	3.58E-05	0.000309	6.756105	7.392797	0.913877	1.06712	Downregulated
**7949273**	-0.16076	3.58E-05	0.000309	8.386471	9.375023	0.894555	1.06712	Downregulated
**8014591**	-0.10195	3.59E-05	0.000309	8.134524	8.730158	0.931773	1.06712	Downregulated
**8032789**	-0.10381	3.82E-05	0.000326	8.559453	9.198079	0.93057	1.06712	Downregulated
**8046906**	-0.16999	3.88E-05	0.000331	6.881374	7.741879	0.888851	1.06712	Downregulated
**8155898**	-0.15106	3.93E-05	0.000334	8.95438	9.942827	0.900587	1.06712	Downregulated
**7943530**	-0.13805	3.94E-05	0.000334	5.399423	5.941611	0.908747	1.06712	Downregulated
**8088397**	-0.14067	4.11E-05	0.000346	7.451629	8.2148	0.907098	1.06712	Downregulated
**8003578**	-0.20302	4.18E-05	0.000351	9.632344	11.08787	0.868728	1.06712	Downregulated
**8114491**	-0.26356	4.19E-05	0.000352	7.949241	9.542543	0.833032	1.06712	Downregulated

**7953590**	-0.11438	4.21E-05	0.000353	6.871227	7.438195	0.923776	1.06712	Downregulated
**8079966**	-0.12291	4.40E-05	0.000366	7.955403	8.662859	0.918335	1.06712	Downregulated
**7972692**	-0.37001	4.44E-05	0.000369	6.868466	8.87656	0.773776	1.06712	Downregulated
**7983650**	-0.0974	4.45E-05	0.000369	9.255226	9.901639	0.934717	1.06712	Downregulated
**8137271**	-0.09572	4.55E-05	0.000376	9.726127	10.39333	0.935805	1.06712	Downregulated
**7993126**	-0.11256	4.78E-05	0.000391	7.762668	8.392566	0.924946	1.06712	Downregulated
**8075659**	-0.10356	4.84E-05	0.000395	6.563891	7.052365	0.930736	1.06712	Downregulated
**7961175**	-0.11336	4.91E-05	0.0004	4.529426	4.899697	0.92443	1.06712	Downregulated
**7894781**	-0.16819	4.91E-05	0.0004	3.796044	4.265424	0.889957	1.06712	Downregulated
**8047377**	-0.13082	4.98E-05	0.000404	4.637134	5.077284	0.91331	1.06712	Downregulated
**7934271**	-0.24288	5.18E-05	0.000419	7.001817	8.285613	0.845057	1.06712	Downregulated
**7916248**	-0.10488	5.22E-05	0.000422	6.095943	6.555586	0.929885	1.06712	Downregulated
**8136645**	-0.09494	5.30E-05	0.000427	5.319318	5.681139	0.936312	1.06712	Downregulated
**7894472**	-0.16612	5.32E-05	0.000428	4.553741	5.109454	0.891238	1.06712	Downregulated
**8020827**	-0.45244	5.42E-05	0.000434	7.714101	10.55563	0.730804	1.06712	Downregulated
**7932326**	-0.36355	5.46E-05	0.000437	6.467123	8.320498	0.777252	1.06712	Downregulated
**7938951**	-0.17746	6.09E-05	0.000479	6.021985	6.810207	0.884259	1.06712	Downregulated

**8056363**	-0.09676	6.24E-05	0.000489	6.023754	6.441611	0.935132	1.06712	Downregulated
**7902518**	-0.12638	6.26E-05	0.00049	8.70578	9.502835	0.916124	1.06712	Downregulated
**8155627**	-0.10696	6.27E-05	0.00049	6.000654	6.462466	0.928539	1.06712	Downregulated
**8161381**	-0.10696	6.27E-05	0.00049	6.000654	6.462466	0.928539	1.06712	Downregulated
**7932118**	-0.13523	6.79E-05	0.000523	6.958496	7.642299	0.910524	1.06712	Downregulated
**7945321**	-0.10351	6.88E-05	0.000529	7.827688	8.409957	0.930764	1.06712	Downregulated
**7918622**	-0.10813	6.98E-05	0.000536	7.661612	8.257913	0.92779	1.06712	Downregulated
**7922994**	-0.29325	6.99E-05	0.000536	6.181826	7.5752	0.816061	1.06712	Downregulated
**8180391**	-0.0982	7.10E-05	0.000544	7.912221	8.469552	0.934196	1.06712	Downregulated
**7996563**	-0.1238	7.47E-05	0.000567	9.641671	10.50556	0.917769	1.06712	Downregulated
**8160040**	-0.10134	7.47E-05	0.000567	7.6096	8.163362	0.932165	1.06712	Downregulated
**7970455**	-0.10831	8.08E-05	0.000605	8.804271	9.490697	0.927674	1.06712	Downregulated
**8074335**	-0.19401	8.20E-05	0.000613	6.522568	7.461437	0.874171	1.06712	Downregulated
**8145227**	-0.10461	8.21E-05	0.000613	7.449068	8.009239	0.930059	1.06712	Downregulated
**7907286**	-0.33747	8.21E-05	0.000614	5.252813	6.637153	0.791426	1.06712	Downregulated
**8131927**	-0.13034	8.48E-05	0.00063	6.918376	7.572543	0.913613	1.06712	Downregulated
**7948982**	-0.19482	8.73E-05	0.000646	7.451994	8.529413	0.873682	1.06712	Downregulated

**8152719**	-0.1557	9.13E-05	0.000669	6.545486	7.291427	0.897696	1.06712	Downregulated
**8167356**	-0.10764	9.13E-05	0.000669	6.66022	7.176154	0.928104	1.06712	Downregulated
**8007429**	-0.38786	9.20E-05	0.000673	6.14665	8.042606	0.764261	1.06712	Downregulated
**8060997**	-0.12811	9.24E-05	0.000676	7.31282	7.991921	0.915027	1.06712	Downregulated
**8155508**	-0.1112	9.25E-05	0.000676	4.024232	4.346679	0.925818	1.06712	Downregulated
**8095390**	-0.13773	9.27E-05	0.000678	5.834114	6.418549	0.908946	1.06712	Downregulated
**7982341**	-0.13929	9.66E-05	0.000701	7.616446	8.388476	0.907965	1.06712	Downregulated
**7921031**	-0.11401	9.85E-05	0.000711	5.373839	5.81573	0.924018	1.06712	Downregulated
**8076007**	-0.09778	9.95E-05	0.000717	8.504085	9.100415	0.934472	1.06712	Downregulated
**8016482**	-0.10468	0.000104	0.00074	7.072171	7.604369	0.930014	1.06712	Downregulated
**8055992**	-0.10607	0.000104	0.000742	7.892691	8.494857	0.929114	1.06712	Downregulated
**8127079**	-0.10312	0.000104	0.000742	4.972089	5.340479	0.931019	1.06712	Downregulated
**8155418**	-0.09424	0.000104	0.000746	5.561905	5.937338	0.936767	1.06712	Downregulated
**8155591**	-0.1706	0.000105	0.00075	5.966803	6.715816	0.88847	1.06712	Downregulated
**8098041**	-0.10918	0.000108	0.000767	7.859273	8.477122	0.927116	1.06712	Downregulated
**8108006**	-0.24978	0.000109	0.000771	6.946164	8.259148	0.841027	1.06712	Downregulated
**8051215**	-0.16618	0.000109	0.000775	6.795216	7.624793	0.8912	1.06712	Downregulated

**8044080**	-0.17199	0.000111	0.000787	8.396615	9.459731	0.887617	1.06712	Downregulated
**8002249**	-0.11737	0.000113	0.000801	8.962356	9.721975	0.921866	1.06712	Downregulated
**8155167**	-0.09586	0.000121	0.000846	7.502559	8.018025	0.935712	1.06712	Downregulated
**7893920**	-0.10962	0.000125	0.000867	6.007465	6.481729	0.926831	1.06712	Downregulated
**7991800**	-0.09738	0.000127	0.000879	6.833181	7.310344	0.934728	1.06712	Downregulated
**8097920**	-0.20762	0.000128	0.000885	5.762787	6.654773	0.865963	1.06712	Downregulated
**7923628**	-0.12406	0.000129	0.000893	7.001494	7.630218	0.917601	1.06712	Downregulated
**8103094**	-0.09838	0.000131	0.000904	8.81261	9.43451	0.934082	1.06712	Downregulated
**7919243**	-0.17808	0.000132	0.000912	5.344231	6.046335	0.883879	1.06712	Downregulated
**8039389**	-0.09781	0.000134	0.000919	9.233628	9.881347	0.93445	1.06712	Downregulated
**8080762**	-0.11057	0.000134	0.00092	8.373498	9.040469	0.926224	1.06712	Downregulated
**7937016**	-0.10629	0.000136	0.000933	9.052324	9.744426	0.928975	1.06712	Downregulated
**7956120**	-0.09681	0.000137	0.000936	9.364332	10.0143	0.935096	1.06712	Downregulated
**8138745**	-0.10417	0.000141	0.00096	7.977804	8.575158	0.930339	1.06712	Downregulated
**8103769**	-0.10633	0.000142	0.000967	10.30938	11.09792	0.928946	1.06712	Downregulated
**8100784**	-0.09947	0.000143	0.000972	4.700969	5.036522	0.933376	1.06712	Downregulated
**7904408**	-0.25895	0.000144	0.000974	5.859178	7.01114	0.835695	1.06712	Downregulated

**7913768**	-0.09703	0.000144	0.000978	8.219114	8.790888	0.934958	1.06712	Downregulated
**7942603**	-0.17317	0.000146	0.000989	8.898438	10.03331	0.88689	1.06712	Downregulated
**8018006**	-0.09946	0.000149	0.001004	5.178262	5.547865	0.933379	1.06712	Downregulated
**8030148**	-0.16598	0.000155	0.001039	6.9519	7.799556	0.89132	1.06712	Downregulated
**8101874**	-0.11813	0.000159	0.00106	7.186842	7.8001	0.921378	1.06712	Downregulated
**7973869**	-0.10543	0.000166	0.001101	4.989584	5.367859	0.92953	1.06712	Downregulated
**7970793**	-0.11219	0.000167	0.001102	9.362967	10.12014	0.925182	1.06712	Downregulated
**7904843**	-0.34341	0.000169	0.001113	6.064106	7.693857	0.788175	1.06712	Downregulated
**7993815**	-0.10152	0.000178	0.001162	8.79289	9.43389	0.932054	1.06712	Downregulated
**8076424**	-0.10542	0.00018	0.001175	7.477832	8.0447	0.929535	1.06712	Downregulated
**7973530**	-0.13944	0.000183	0.001194	9.251991	10.19083	0.907875	1.06712	Downregulated
**8003939**	-0.15507	0.000187	0.001211	8.399636	9.352797	0.898088	1.06712	Downregulated
**7893525**	-0.11506	0.000187	0.001214	6.927953	7.503094	0.923346	1.06712	Downregulated
**8083978**	-0.10143	0.000191	0.001234	6.870919	7.371354	0.932111	1.06712	Downregulated
**7940323**	-0.24217	0.000208	0.001331	8.323569	9.844871	0.845473	1.06712	Downregulated
**8139640**	-0.14494	0.000209	0.001338	8.444215	9.336654	0.904416	1.06712	Downregulated
**7987012**	-0.09678	0.000214	0.001362	7.210459	7.710757	0.935117	1.06712	Downregulated

**7901765**	-0.09499	0.000215	0.00137	8.016409	8.561963	0.936282	1.06712	Downregulated
**7908610**	-0.18576	0.000217	0.001377	5.867732	6.674055	0.879185	1.06712	Downregulated
**8009713**	-0.11156	0.000218	0.001383	6.550276	7.076877	0.925589	1.06712	Downregulated
**8160297**	-0.13888	0.000225	0.001421	8.540576	9.403627	0.908221	1.06712	Downregulated
**8133034**	-0.10105	0.000234	0.001469	5.095636	5.465331	0.932356	1.06712	Downregulated
**7964316**	-0.14391	0.000236	0.001481	9.998937	11.04782	0.90506	1.06712	Downregulated
**8154447**	-0.19102	0.000239	0.001494	5.688232	6.493539	0.875983	1.06712	Downregulated
**8074716**	-0.101	0.000242	0.001509	8.416773	9.027117	0.932388	1.06712	Downregulated
**8088474**	-0.10714	0.000254	0.001572	5.523573	5.949384	0.928428	1.06712	Downregulated
**7961390**	-0.14854	0.000259	0.001601	8.911868	9.878358	0.902161	1.06712	Downregulated
**7895105**	-0.10474	0.000275	0.001691	7.283774	7.832227	0.929975	1.06712	Downregulated
**8081488**	-0.1156	0.000283	0.001732	10.48831	11.36327	0.923001	1.06712	Downregulated
**8101893**	-0.17118	0.000291	0.001775	9.537753	10.73928	0.888119	1.06712	Downregulated
**8122071**	-0.1745	0.000298	0.001809	8.381867	9.45957	0.886073	1.06712	Downregulated
**7916075**	-0.13461	0.000308	0.001865	3.907463	4.289587	0.910918	1.06712	Downregulated
**7894202**	-0.10899	0.000311	0.00188	5.49462	5.925811	0.927235	1.06712	Downregulated
**8074316**	-0.10171	0.000314	0.001892	8.620989	9.250728	0.931925	1.06712	Downregulated

**7961455**	-0.1143	0.000319	0.001916	8.789723	9.514453	0.923828	1.06712	Downregulated
**7991335**	-0.17808	0.00032	0.001919	11.22738	12.70243	0.883877	1.06712	Downregulated
**8105899**	-0.09394	0.000321	0.001923	8.686423	9.270831	0.936963	1.06712	Downregulated
**8177498**	-0.09394	0.000321	0.001923	8.686423	9.270831	0.936963	1.06712	Downregulated
**7893832**	-0.11541	0.00033	0.00197	5.526854	5.987139	0.923121	1.06712	Downregulated
**7957835**	-0.31289	0.00034	0.002021	6.12428	7.607545	0.805027	1.06712	Downregulated
**8142171**	-0.15592	0.000344	0.002039	11.31068	12.60161	0.897558	1.06712	Downregulated
**8067963**	-0.14034	0.000349	0.002068	5.462647	6.020755	0.907303	1.06712	Downregulated
**8155414**	-0.16175	0.000359	0.002118	5.827178	6.518529	0.893941	1.06712	Downregulated
**8161384**	-0.16175	0.000359	0.002118	5.827178	6.518529	0.893941	1.06712	Downregulated
**8016463**	-0.10839	0.000381	0.002226	7.408558	7.986607	0.927623	1.06712	Downregulated
**8053737**	-0.14812	0.00043	0.002459	5.518672	6.115393	0.902423	1.06712	Downregulated
**7955702**	-0.16925	0.000437	0.002491	6.608786	7.431419	0.889303	1.06712	Downregulated
**8041061**	-0.25345	0.000452	0.00256	7.321133	8.727217	0.838885	1.06712	Downregulated
**8005879**	-0.22332	0.000456	0.002581	8.280301	9.666593	0.856589	1.06712	Downregulated
**8136652**	-0.09601	0.000458	0.00259	5.086642	5.436686	0.935614	1.06712	Downregulated
**8097809**	-0.11674	0.000461	0.002602	5.647514	6.123501	0.922269	1.06712	Downregulated

**8124897**	-0.11929	0.000464	0.002615	5.479725	5.952094	0.920638	1.06712	Downregulated
**8179727**	-0.11929	0.000464	0.002615	5.479725	5.952094	0.920638	1.06712	Downregulated
**8146986**	-0.16604	0.000469	0.002636	9.247098	10.37503	0.891284	1.06712	Downregulated
**8027354**	-0.1411	0.000474	0.002657	5.222145	5.758682	0.90683	1.06712	Downregulated
**8053654**	-0.12107	0.000494	0.00275	9.787085	10.64387	0.919505	1.06712	Downregulated
**7962083**	-0.11986	0.000499	0.002772	7.228615	7.854826	0.920277	1.06712	Downregulated
**7924401**	-0.31189	0.000503	0.002791	5.929749	7.360806	0.805584	1.06712	Downregulated
**8008564**	-0.11049	0.000519	0.002863	6.403851	6.913567	0.926273	1.06712	Downregulated
**7895529**	-0.10948	0.000526	0.002892	4.283528	4.621234	0.926923	1.06712	Downregulated
**8169836**	-0.30613	0.000527	0.002895	8.51949	10.53337	0.80881	1.06712	Downregulated
**8047829**	-0.35373	0.000531	0.002914	6.239011	7.972592	0.782558	1.06712	Downregulated
**8024754**	-0.29383	0.000545	0.002978	8.30696	10.18343	0.815733	1.06712	Downregulated
**8172204**	-0.22282	0.000546	0.00298	7.437966	8.680182	0.856891	1.06712	Downregulated
**8148331**	-0.10623	0.000563	0.003056	3.850761	4.145005	0.929012	1.06712	Downregulated
**7947165**	-0.37556	0.000579	0.003125	6.895948	8.946414	0.770806	1.06712	Downregulated
**8110616**	-0.24737	0.000638	0.003391	6.08464	7.222723	0.84243	1.06712	Downregulated
**7903742**	-0.11337	0.000648	0.00343	9.476509	10.25127	0.924423	1.06712	Downregulated

**7909866**	-0.1175	0.000677	0.003566	7.676183	8.327532	0.921784	1.06712	Downregulated
**8104268**	-0.29264	0.000697	0.00365	8.490478	10.3998	0.816408	1.06712	Downregulated
**8048432**	-0.147	0.000704	0.003678	8.115363	8.985878	0.903124	1.06712	Downregulated
**8127087**	-0.13007	0.000707	0.003693	5.180698	5.669489	0.913786	1.06712	Downregulated
**7910948**	-0.09772	0.000722	0.003754	3.680241	3.938168	0.934506	1.06712	Downregulated
**7924403**	-0.27233	0.000729	0.003785	5.883127	7.105404	0.827979	1.06712	Downregulated
**8026456**	-0.10216	0.000761	0.003931	7.59895	8.156542	0.931639	1.06712	Downregulated
**8083743**	-0.10669	0.000788	0.004051	7.676745	8.265979	0.928716	1.06712	Downregulated
**8013989**	-0.31248	0.000798	0.004092	6.978036	8.6656	0.805257	1.06712	Downregulated
**8117288**	-0.09792	0.000834	0.004242	5.714476	6.115791	0.934381	1.06712	Downregulated
**8071861**	-0.19942	0.000837	0.004255	8.104212	9.305549	0.870901	1.06712	Downregulated
**7895154**	-0.11937	0.000853	0.004325	6.197253	6.731829	0.92059	1.06712	Downregulated
**8047563**	-0.10661	0.000868	0.004388	4.374733	4.710241	0.928771	1.06712	Downregulated
**7893166**	-0.10837	0.000891	0.004478	5.037518	5.430481	0.927637	1.06712	Downregulated
**7985636**	-0.18914	0.000899	0.004513	6.375707	7.268846	0.877128	1.06712	Downregulated
**7910466**	-0.11239	0.000915	0.004576	7.284932	7.87516	0.925052	1.06712	Downregulated
**7951077**	-0.0969	0.000927	0.004625	7.392113	7.905687	0.935037	1.06712	Downregulated

**7893144**	-0.12669	0.000932	0.004646	3.911424	4.270451	0.915928	1.06712	Downregulated
**7996331**	-0.17104	0.000934	0.004653	7.542923	8.492363	0.888201	1.06712	Downregulated
**8094361**	-0.30705	0.000943	0.004687	7.264306	8.987192	0.808295	1.06712	Downregulated
**8037949**	-0.32161	0.000982	0.004839	5.400604	6.749287	0.800174	1.06712	Downregulated
